# Antibiotics may increase triazine herbicide exposure risk via disturbing gut microbiota

**DOI:** 10.1186/s40168-018-0602-5

**Published:** 2018-12-13

**Authors:** Jing Zhan, Yiran Liang, Donghui Liu, Xiaoran Ma, Peize Li, Chang Liu, Xueke Liu, Peng Wang, Zhiqiang Zhou

**Affiliations:** Beijing Advanced Innovation Center for Food Nutrition and Human Health, College of Science, China Agricultural University, No. 2, West Yuanmingyuan Road, Beijing, 100193 People’s Republic of China

**Keywords:** Antibiotics, Pesticide exposure, Gut microbiota, Bioavailability

## Abstract

**Background:**

Antibiotics are commonly used worldwide, and pesticide is a kind of xenobiotic to which humans are frequently exposed. The interactive impact of antibiotics on pesticides has rarely been studied. We aim to investigate the effects of antibiotics on the pesticide exposure risk and whether gut microbiota altered by antibiotics has an influence on pesticide bioavailability. Furthermore, we explored the mechanisms of gut microbiota affecting the fate of pesticides in the host.

**Results:**

The oral bioavailability of triazine herbicides significantly increased in the rats treated with ampicillin or antibiotic cocktails. The antibiotic-altered gut microbiota directly influenced the increased pesticide bioavailability through downregulating hepatic metabolic enzyme gene expression and upregulating intestinal absorption-related proteins.

**Conclusions:**

Antibiotics could increase the pesticide bioavailability and thereby may increase the pesticide exposure risk. The antibiotic-altered gut microbiota that could alter the hepatic metabolic enzyme gene expression and intestinal absorption-related proteome was a critical cause of the increased bioavailability. This study revealed an undiscovered potential health impact of antibiotics and reminded people to consider the co-exposed xenobiotics when taking antibiotics.

**Electronic supplementary material:**

The online version of this article (10.1186/s40168-018-0602-5) contains supplementary material, which is available to authorized users.

## Introduction

Pesticides are indispensable for modern agricultural production. A considerable amount of research has shown that pesticide residues could move through the food chain and ultimately pose a health threat to humans. After the mid-1980s, herbicides became the most widely used pesticide globally [1]. In particular, triazine herbicides, such as atrazine and simazine, have been widely applied in agricultural settings and were frequently detected in food, natural water, and soil [2–4]. Triazine herbicides could cause disruption of the endocrine system. For instance, atrazine was regarded as a potential carcinogen that caused hormonal disorders and affected the normal mammalian reproductive function [5–7]. Likewise, exposure to simazine resulted in neonatal birth defects and the potential for developmental disorders [8]. Currently, studies of pesticides have largely focused on the effects of a single pesticide exposure. However, pesticide exposure is likely to result from various sources (e.g. food and water) and occurs simultaneously via multiple pesticide agents [9, 10]. Co-exposure to multiple pesticides may lead to more negative effects.

Antibiotics are one of the greatest discoveries in the history of human medicine, and they play a major role in controlling disease and decreasing pathogen-associated mortality. Many infectious diseases cannot be treated without antibiotics, and the widespread application of antibiotics has led to large levels of human consumption. For example, approximately 3440 and 3290 tons of antibiotics were respectively consumed by Europeans in 2003 and Americans during 2010–2011, while consumption reached 77,760 tons in China in 2013 [11]. Moreover, due to the common use of antibiotics, antibiotic residues have been detected in livestock, companion animals, and even wildlife [12–16]. Residual antibiotics in livestock products such as beef, chicken, and milk might transmit to humans via the consumption of food [17]. The use of antibiotics directly affects gut microbiota by reducing abundance, altering community structure, and reducing bacterial diversity [18, 19]. A growing body of research has elucidated the expansive roles that gut microbiota played in human health. Gut microbiota was related to many diseases, including diabetes, inflammatory bowel disease, autism, liver disease, and cancer [20–26]. Further, gut microbiota could affect the drug metabolism and toxicity in the host [27]. For example, *Eggerthella lenta* could metabolize the cardiac drug digoxin and reduce the drug efficiency [28]. Moreover, gut microbiota could modify the metabolism of acetaminophen and affect the metabolite composition [29]. Thus, antibiotic-altered gut microbiota may affect the chemical transformation of xenobiotics in the host. However, current reports on the effects of antibiotics on xenobiotic metabolism have mostly focused on interactions between antibiotics and drug, while studies on environmental pollutants were rare. Specifically, we have minimal knowledge regarding the impact of antibiotics on the metabolism of pesticides, xenobiotics to which humans are directly or indirectly exposed. When people are treated with antibiotics and exposed to pesticides at the same time, the fate of the pesticides is unknown. Moreover, whether the gut microbiota is involved in this process is unclear.

Oral bioavailability is defined as the rate and extent to which an active moiety is absorbed from a xenobiotic form and becomes available in the systemic circulation [30]. Oral bioavailability, as an important pharmacokinetic property for oral xenobiotics such as drugs, is evaluated by measuring the area under curve (AUC) of a dose-time curve. For pesticides, high bioavailability means the high risks of the increased pesticides entering blood circulation, tissues, and organs. In this study, we assessed the impact of antibiotics on the bioavailability of five typical triazine herbicides (simazine, atrazine, ametryn, terbuthylazine, and metribuzin) after oral exposure in rats. Rats were first treated with a commonly used antibiotic, ampicillin, at a dosage equivalent to that in humans. Ampicillin administration significantly increased the oral bioavailability of triazine herbicides. We then obtained similar results in the antibiotic cocktail-treated rats. By first treating rats with antibiotics, followed by a transfer of microbiota, we successfully constructed a microbiota-deficient model to confirm that gut microbiota had effects on the increased pesticide bioavailability. Mechanisms of gut microbiota affecting the bioavailability were investigated from the perspective of hepatic metabolism and intestinal absorption. These results provided critical data on the xenobiotic metabolism affected by gut microbiota and contributed to a more comprehensive understanding about the effects of antibiotics on pesticide exposure risk.

## Results

### Effect of ampicillin on the triazine herbicide bioavailability

**Fig. 1:**
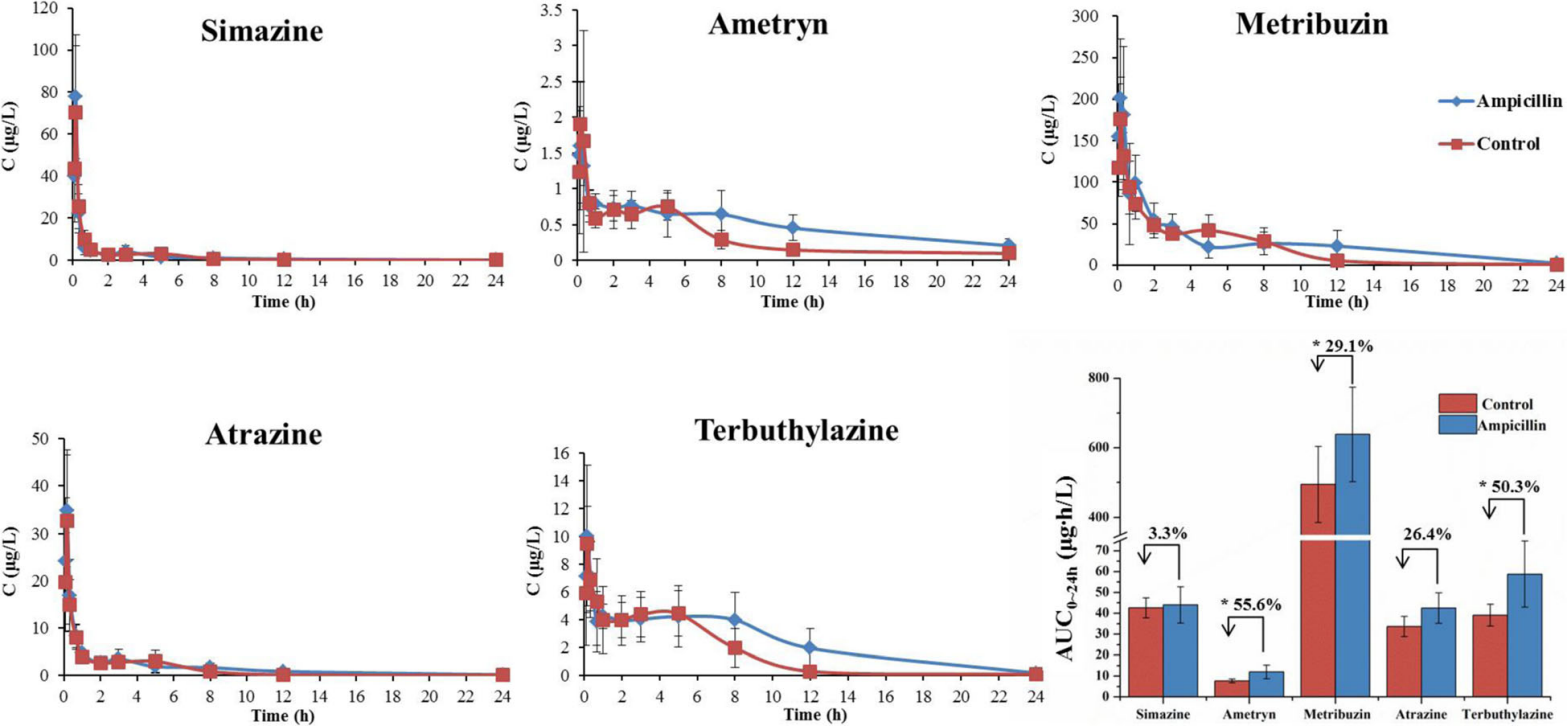
Effects of 3-day ampicillin treatment on the blood concentration of triazine herbicides in rats. Control rats were treated with equal volumes of water free from ampicillin. The AUC of triazine herbicides was showed in the column diagram, and the percent represents the increase rate of the AUC in the ampicillin-treated rats relative to the control rats (independent sample *t*-test, *P < 0.05, *n* = 5)

**Fig. 2:**
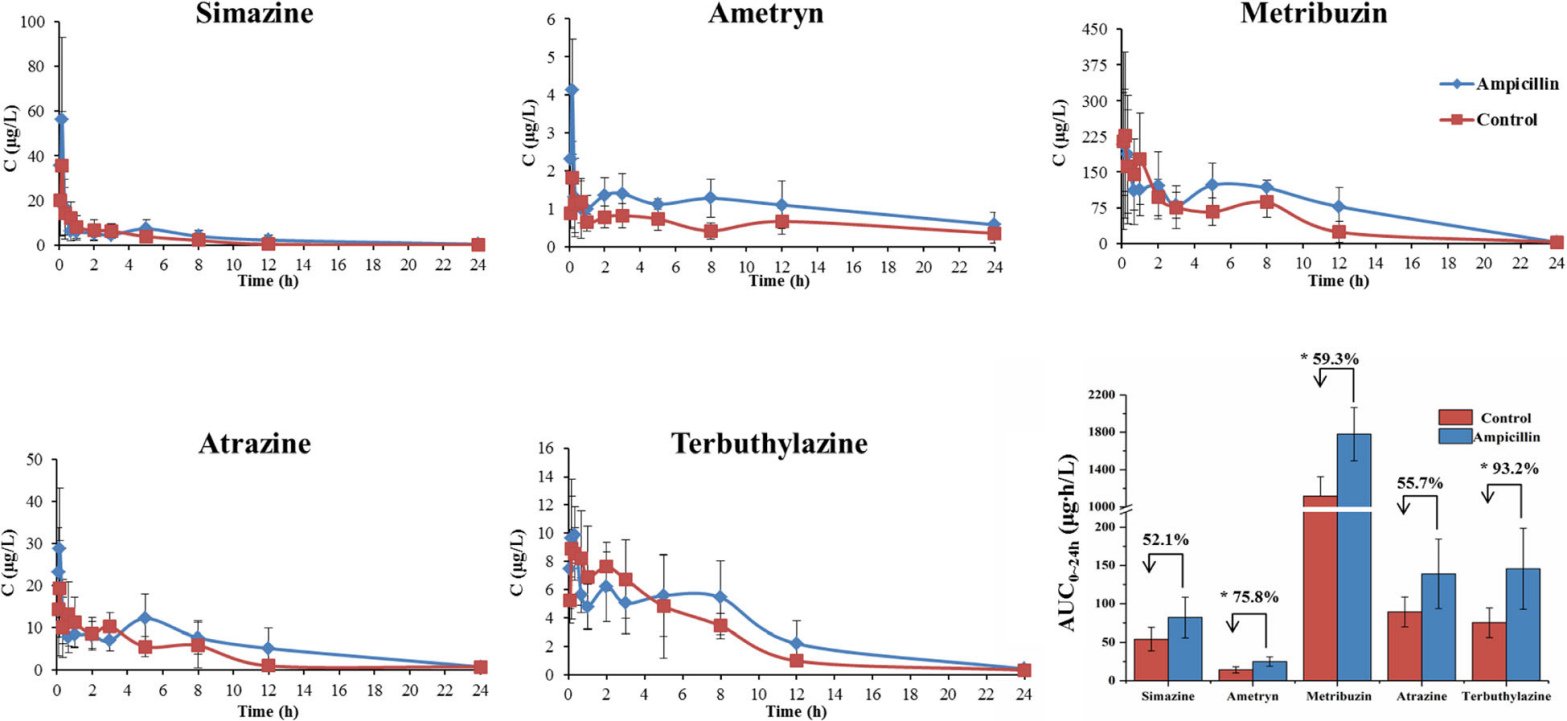
Effects of 7-day ampicillin treatment on the blood concentration of triazine herbicides in rats. Control rats were treated with equal volumes of water free from ampicillin. The AUC of triazine herbicides was showed in the column diagram, and the percent represents the increase rate of the AUC in the ampicillin-treated rats relative to the control rats (independent sample *t*-test, *P < 0.05, *n =* 5)

Rats were treated with the ampicillin dosage converted from the human dosing regimen (gavage of ampicillin three times per day, 90 mg/kg body weight in each dose). After 3 days of ampicillin treatment, the triazine herbicides (2 mg/kg body weight per herbicide in the mixture) were orally administered. The rats were then given ampicillin for four more days, followed by another exposure to triazine herbicides (experimental design presented in Additional file 1: Figure S1). Rats in the control group were treated with ampicillin-free water. After exposure to triazine herbicides, blood samples of rats were collected and tested for herbicides. The herbicide concentration into blood peaked at approximately 10 min in both the ampicillin-treated and control groups, suggesting rapid absorption of triazine herbicides (Fig. 1). The maximum concentrations (*C*_max_) of the herbicides were higher in the ampicillin-treated group compared with the control group (Additional file 1: Table S1). The area under concentration-time curve (AUC), based on simazine, ametryn, metribuzin, atrazine, and terbuthylazine concentrations in blood, increased 3.31%, 55.6%, 29.1%, 26.4%, and 50.3% respectively in the 3-day ampicillin-treated group compared with the control group. Specifically, the AUC of metribuzin, ametryn, and terbuthylazine was significantly higher in the ampicillin treatment group than that in the control group (*P* < 0.05). Similar results were observed after 7 days of oral ampicillin administration (Fig. 2), further indicating that ampicillin treatment could lead to an increase in triazine herbicide bioavailability.

**Fig. 3:**
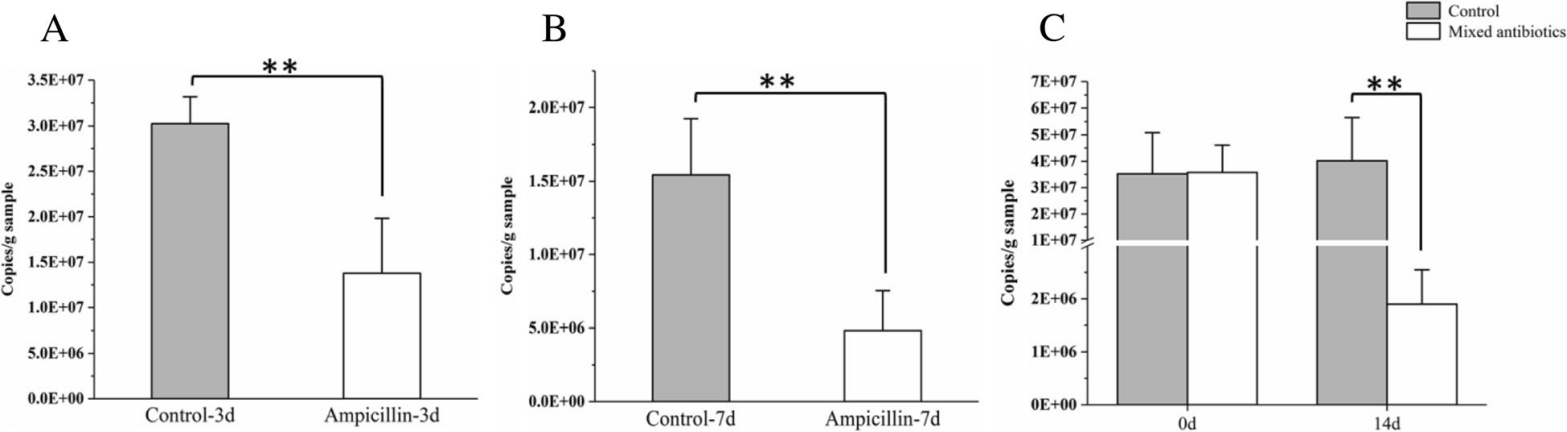
Effects of ampicillin and antibiotic cocktail treatment on the amount of cecal bacteria in rats. Prokaryotic 16S rRNA gene copies in cecal feces samples from 3-day ampicillin (**a**), 7-day ampicillin (**b**), and 14-day antibiotic cocktail (**c**) treated rats (independent sample *t*-test, **P < 0.01, *n =* 5)

In addition, gut microbial amount and composition were measured to observe the effects of ampicillin exposure on gut microbiota. As shown in Fig. 3, both 3-day and 7-day ampicillin treatment significantly decreased bacterial amount in rats (*P* < 0.01). Ampicillin treatment for 3 and 7 days also reduced the Shannon index and led to apparent compositional distinction based on principal coordinate analyses (PCoA) of the Bray-Curtis distances (Additional file 1: Figure S2 and Figure S3). As shown in the heat map based on significantly changed microbial species (Additional file 1: Figure S2C and Figure S3C), the relative abundance of species from the genus such as *Ruminococcaceae*, *Lachnospiraceae*, and *Anaerotruncus* was decreased in the rats treated with 3-day and 7-day ampicillin. These results indicated that ampicillin treatment apparently changed microbial diversity and composition.

### Antibiotic cocktails increase triazine herbicide bioavailability in rats

After confirming that treatment with a single antibiotic resulted in increased bioavailability of triazine herbicides, we further tested whether increased antibiotic doses would result in similar phenomena and whether these processes were associated with the effects arising from the antibiotic-caused gut microbiota disruption. Previous studies have reported that the fecal microbial amount in mice decreased by 90–95% after 14 days of oral treatment with an antibiotics cocktail [31]. In accordance with this method, continuous antibiotic cocktails (ampicillin, neomycin, gentamicin, and metronidazole each at 1.75 mg/day and vancomycin at 0.875 mg/day) were conducted by gavage for 14 days, after which rats were orally exposed to triazine herbicides. Two concentrations of triazine herbicides were used (20 mg/kg and 2 mg/kg body weight per herbicide, respectively) to comprehensively study the effect of antibiotics on herbicide bioavailability. After continuous treatment with antibiotic cocktails for 14 days, the AUC and *C*_max_ in the antibiotic-treated rats were significantly higher than that in the control group, with AUC increased by 39.42–94.92% (20 mg/kg body weight of herbicides, *P* < 0.05, Additional file 1: Figure S4 and Table S2) and 44.78–151.43% (2 mg/kg body weight of herbicides, *P* < 0.05, Additional file 1: Figure S5 and Table S2). Meanwhile, gut microbial amount and composition were measured to observe the effect of antibiotic cocktail exposure on gut microbiota. As shown in Fig. 3, the initial total amount of bacteria was not apparently different, while bacterial amount was significantly decreased after the 14-day antibiotic cocktail treatment compared with the antibiotic untreated rats (*P* < 0.01). Microbial diversity and composition were also apparently altered by 14-day antibiotic cocktail treatment from the results of the Shannon index, PCoA of the Bray-Curtis distances, and species comparison (Additional file 1: Figure S6). The proportion of species from the genus such as *Ruminococcaceae* and *Anaerotruncus* was changed (Additional file 1: Figure S6C).

### In vitro microbial metabolism and microsomal metabolism of triazine herbicides

Gut microbiota could impact xenobiotic metabolism in a variety of ways. Previous studies showed that antibiotics could shape the physiology and gene expression of the active gut microbiome and then affect the microbial metabolism of xenobiotics [32, 33]. To investigate whether gut microbiota from rats exposed to antibiotics affected the triazine herbicide degradation and then caused the increased herbicide bioavailability, metabolism of triazine herbicides by gut microbiota was investigated using fecal bacteria culture in vitro under anaerobic conditions. The initial concentration of triazine herbicides was designed at 2 mg/L and 0.02 mg/L respectively for degradation assay. As shown in Additional file 1: Figure S7 and Figure S8, the triazine herbicides were not metabolized whether in sterilized culture medium without bacteria or bacteria culture from antibiotic-treated and antibiotic-untreated rats. The results indicated that microbial metabolism of herbicides may contribute little to the increased herbicide bioavailability.

Treatments with large amounts of antibiotics could apparently alter gut microbiota, but may also affect the host’s metabolic function. In the antibiotic-treated model, antibiotic intake may directly affect hepatic metabolism in rats, which may also lead to increases in pesticide bioavailability. A liver microsomal model in vitro was used to investigate the effects of antibiotics on hepatic metabolism of triazine herbicides. Liver microsomes account for the most popular in vitro model for xenobiotic metabolism because they are rich in metabolic enzymes such as cytochrome P450 enzymes [34, 35]. Absorption of the antibiotics used in our experiments (ampicillin, neomycin, gentamicin, metronidazole, and vancomycin) was relatively low, and the antibiotic concentrations in rat blood after gavage was 7 mg/L or less. Therefore, 7 mg/L of antibiotic mixture was added with the combination herbicides into the microsome incubation system. The results showed that the antibiotics did not significantly affect the microsomal metabolism of triazine herbicides compared with the control group (Additional file 1: Figure S9). These results suggested that antibiotics might not directly affect triazine herbicide metabolism by inhibiting the enzymes in a short time. Antibiotics may affect triazine herbicide bioavailability via indirect ways.

### Gut microbiota affects triazine herbicide bioavailability

**Fig. 4:**
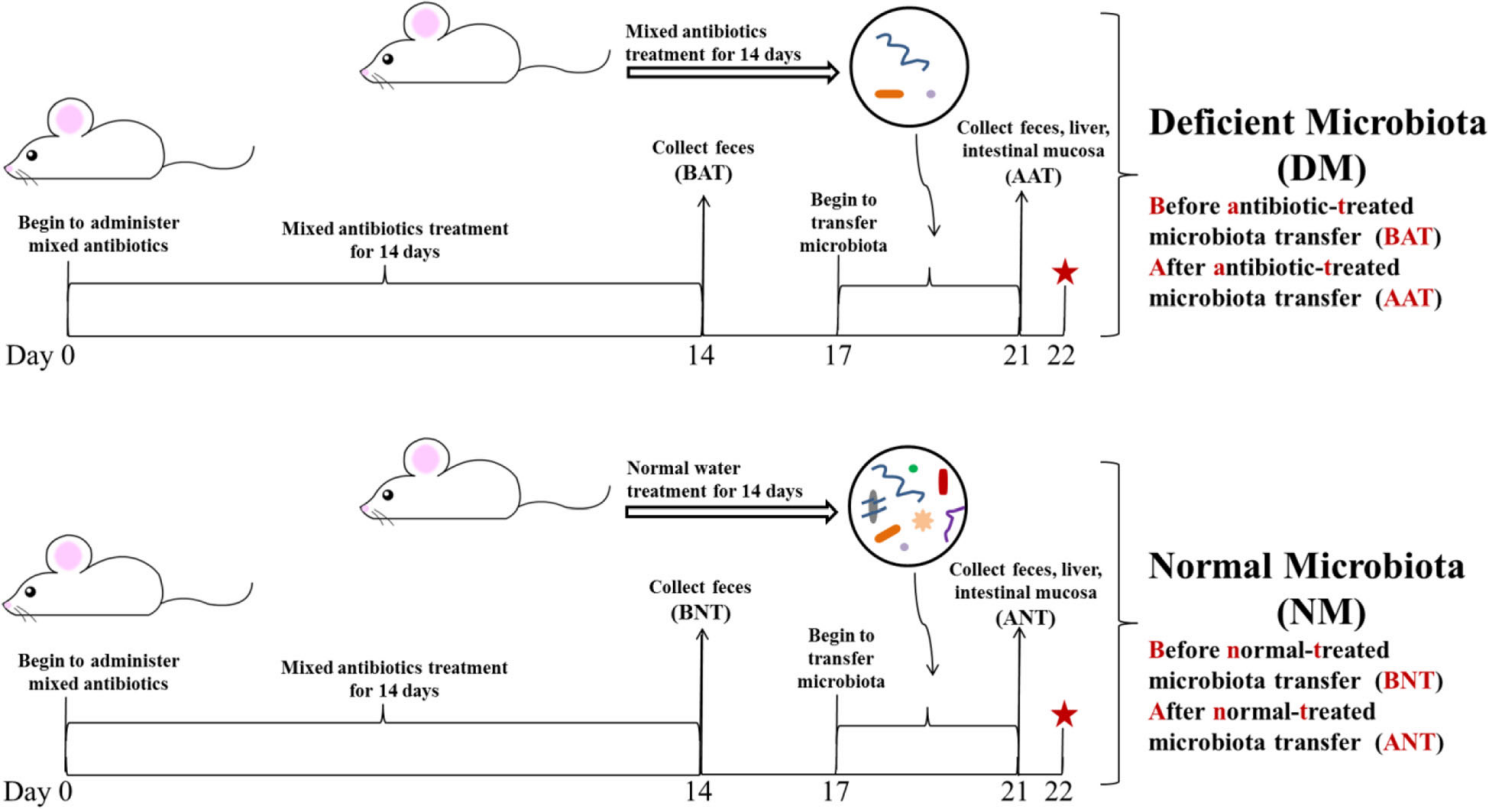
Experimental design of microbiota transfer model based on the antibiotic cocktail-treated rats. Two groups of rats reached a near germ-free state after 14 days of antibiotic cocktail treatment, and these rats were transplanted with gut microbiota from the antibiotic-treated and untreated rats for 4 days to obtain a deficient microbiota group and a normal microbiota group (*n =* 11). Before and after microbiota transplantation, fresh feces of rats was collected, and after microbiota transplantation, rats were euthanized to collect liver and small intestinal mucosa (BAT: before antibiotic-treated microbiota transfer; BNT: before normal-treated microbiota transfer samples; AAT: after antibiotic-treated microbiota transfer; ANT: after normal-treated microbiota transfer, *n* = 5). The remaining rats were used to investigate pesticide bioavailability (*n* = 6). The red star means the beginning of pesticides exposure

**Fig. 5:**
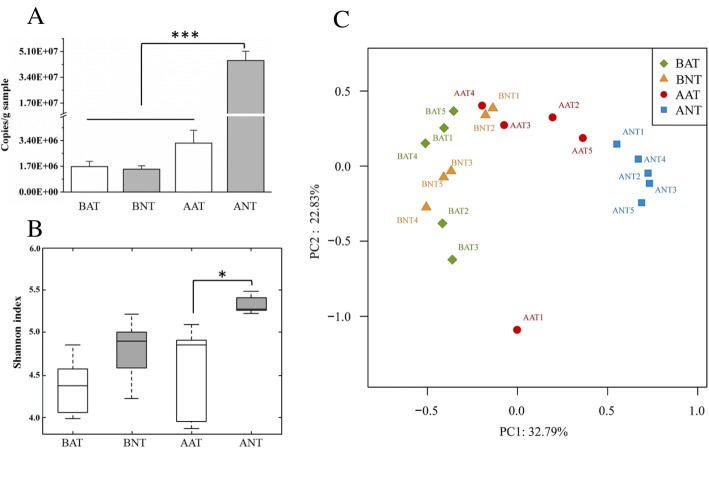
Differences in the gut microbiota of rats before and after microbiota transplantation (BAT: before antibiotic-treated microbiota transfer; BNT: before normal-treated microbiota transfer samples; AAT: after antibiotic-treated microbiota transfer; ANT: after normal-treated microbiota transfer, *n =* 5). **a** Prokaryotic 16S rRNA gene copies in fecal samples (one-way ANOVA followed by Bonferroni’s test, ***P < 0.001). **b** Shannon index for microbial diversity (one-way ANOVA followed by Bonferroni’s test, *P < 0.05). **c** Principal coordinate analyses (PCoA) of the Bray-Curtis calculation showing microbial composition dissimilarity among BAT, BNT, AAT, and ANT samples

The effects of gut microbiota on triazine herbicide bioavailability could not be directly assessed in the antibiotic-treated rats. Consequently, we designed an experiment to investigate these effects with the presence of gut microbiota as the only variable. As shown in Fig. 4, two groups of rats reached a nearly germ-free state after 14 days of antibiotic cocktail treatment. Following a 3-day washout period, gut microbiota from antibiotic-treated and antibiotic-untreated rats was transferred to these rats for four continuous days to obtain a deficient microbiota group (DM group) and a normal microbiota group (NM group). All rats were then orally exposed to 2 mg/kg body weight of triazine herbicides. Fecal samples were respectively collected before antibiotic-treated microbiota transfer (BAT) and normal-treated microbiota transfer (BNT) as well as after antibiotic-treated microbiota transfer (AAT) and normal-treated microbiota transfer (ANT) to assess the abundance and composition of gut microbiota. Total bacteria levels had no significant differences and microbial diversity was similar between the two groups before microbiota transplantation (Fig. 5a and Fig. 5b). After microbiota transplantation, the microbial amount significantly increased in the normal microbiota rats while the microbial amount in the deficient microbiota group did not change significantly. The abundance of total fecal bacteria in the normal microbiota rats was nearly 9 times higher than that in the deficient microbiota rats (*P* < 0.001, Fig. 5a). Microbial diversity also showed an increase in the normal microbiota rats compared with the deficient microbiota rats based on the Shannon index (Fig. 5b). Meanwhile, PCoA of the Bray-Curtis and UniFrac comparison presented that microbial composition of the normal microbiota rats varied from the deficient microbiota rats (Fig. 5c and Additional file 1: Figure S10). LEfSe analysis showed that the proportion of phylum *Firmicutes* and class *Coriobacteriia* in the normal microbiota rats was significantly higher than in the deficient microbiota rats (*P* < 0.05, Additional file 1: Figure S11). At a species level, the proportion of species from the genus such as *Lachnospiraceae*, *Ruminococcaceae*, and *Oscillibacter* was significantly reduced in the deficient microbiota rats relative to involved the normal microbiota rats (*P* < 0.05, Additional file 1: Figure S12). Therefore, considerable differences existed between floral characteristics of the deficient microbiota and normal microbiota groups, suggesting that the microbiota transfer model was successfully developed to confirm the impact of gut microbiota on the herbicide bioavailability.

**Fig. 6:**
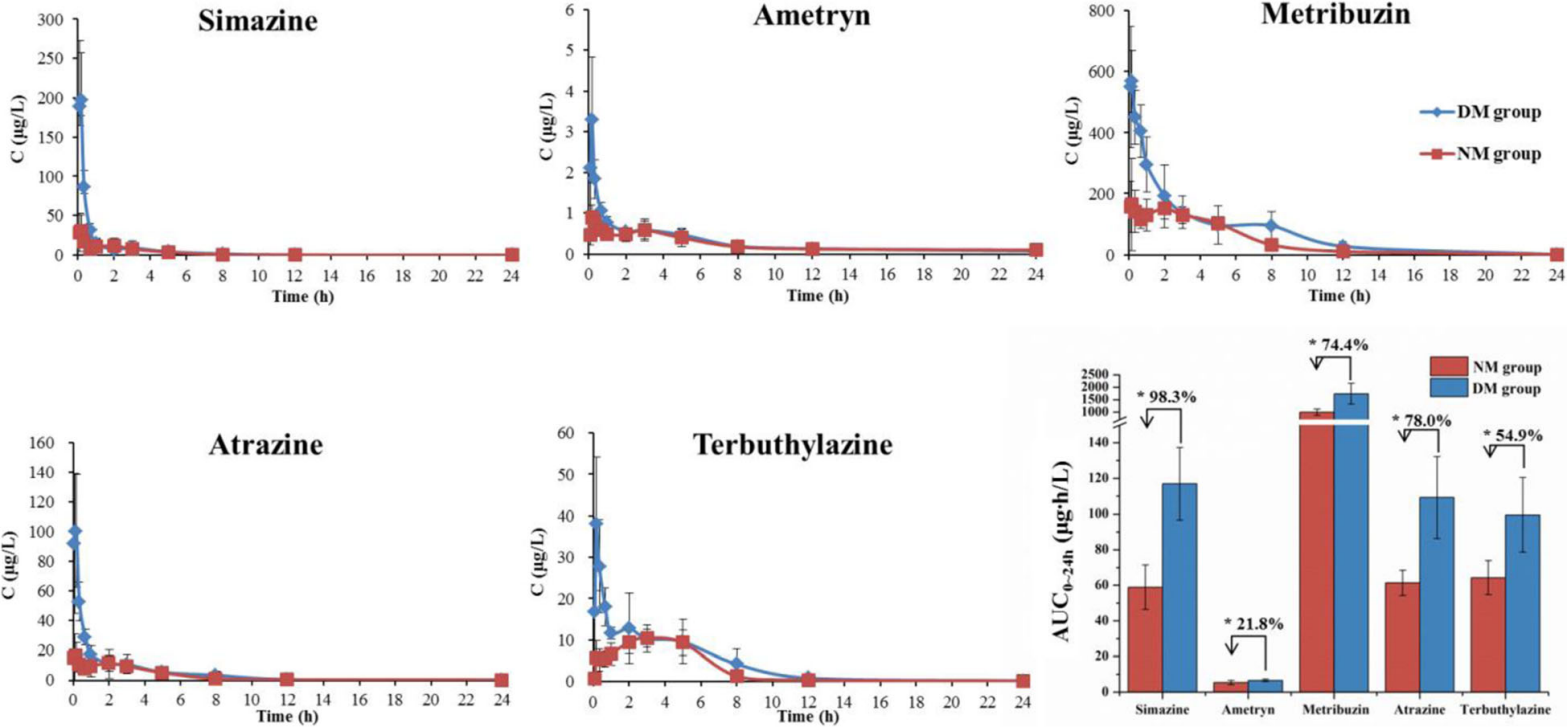
Effects of the gut microbiota on the blood concentration of triazine herbicides in rats. The AUC of triazine herbicides were showed in the column diagram, and the percent means the increase rate of the AUC in the deficient microbiota rats relative to the normal microbiota rats (DM group: the deficient microbiota rats; NM group: the normal microbiota rats; independent sample *t*-test, *P < 0.05, *n =* 6)

Remarkably, the AUC of triazine herbicides was all significantly higher in the deficient microbiota rats than that in the normal microbiota rats (*P* < 0.05, Fig. 6 and Additional file 1: Table S3). The results showed that the gut microbiota deficiency increased the oral bioavailability of triazine herbicides in rats, thus directly indicating that gut microbiota could affect the pesticide bioavailability.

### Gut microbiota alters hepatic metabolism and intestinal absorption-related proteome

To determine the mechanisms of gut microbiota affecting the bioavailability of triazine herbicides, we analyzed the expression of hepatic metabolic enzymes and conducted proteomics on the small intestinal mucosa of rats after microbiota transplantation.

**Fig. 7:**
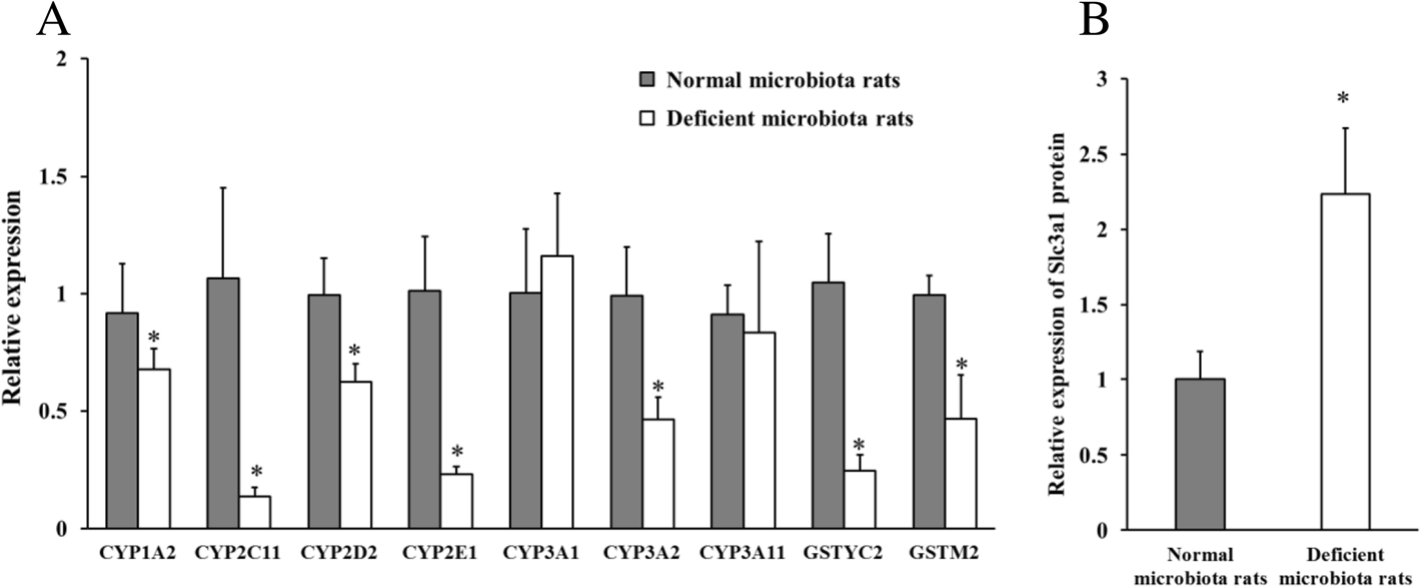
**a** Relative mRNA expression of hepatic metabolic enzymes in the deficient microbiota rats and the normal microbiota rats. **b** Relative expression of Slc3a1 protein in small intestinal mucosa in the deficient microbiota rats and the normal microbiota rats (independent sample *t*-test, *P < 0.05, *n =* 5)

The hepatic enzymes including cytochrome P450 enzymes (CYPs) and glutathione transferase enzymes were involved in the triazine herbicide metabolism, and their activity was also verified in a hepatic microsomal metabolism assay by observing the inhibition of triazine herbicide metabolism when relevant enzyme inhibitors were added (Additional file 1: Figure S13). Compared to the normal microbiota rats, hepatic mRNA expression of CYP1A2, CYP2C11, CYP2D2, CYP2E1, CYP3A2, CYP3A11, GSTYc2, and GSTM2 enzymes was lower in the deficient microbiota rats (Fig. 7a). These results suggested that triazine herbicide metabolism may be reduced because of the downregulation of the metabolic enzymes, and this helped explain why the bioavailability increased in the deficient microbiota rats.

The uptake of orally introduced xenobiotics primarily occurs in small intestine. Label-free proteomics analysis was applied to characterize the effect of gut microbiota on the intestinal absorption of xenobiotics (protein classification in Additional file 1: Figure S14). A total of 77 significantly different proteins were identified in mucosa of the terminal ileum by comparing the deficient microbiota rats and the normal microbiota rats, and these proteins were showed in Additional file 1: Table S4. Of these proteins, 14 proteins were decreased, while the other 63 proteins were increased in the deficient microbiota rats relative to the normal microbiota rats. Among the changed proteins, Slc3a1 (accession number Q64319), a kind of protein proved to have capacity for xenobiotics transport, was upregulated in the deficient microbiota rats compared with the normal microbiota rats (*P* < 0.05, Fig. 7b). Significant changes in other proteins that are associated with drug absorption and transport were not apparent. Thus, the effect of gut microbiota deficiency on xenobiotic absorption may not be significant, and the pathway that gut microbiota promoted the intestinal absorption by upregulating transport protein may be not the most crucial reason for the increased bioavailability in the deficient microbiota rats.

## Discussion

The global use of antibiotics makes it difficult to avoid interaction with other xenobiotics. Previous studies have reported the effects on drug metabolism by antibiotics and antibiotic-altered microbiome. Kim et al. found that oral bioavailability of the hypolipidemic drug lovastatin was decreased in concert with the reduced hydroxyl acid metabolites after antibiotic administration [36]. After oral administration of ampicillin for 3 days, the bioavailability of the antihypertensive drug amlodipine was significantly increased in rats, and the metabolism of amlodipine was reduced by the antibiotic-altered microbiota in in vitro floral culture experiments [37]. Jeong et al. also found that the oral bioavailability of baicalin was decreased and the metabolite profiles changed when gut microbiota was disturbed [38]. Here, we first found that the triazine herbicide bioavailability was increased in rats after 3 and 7 days of ampicillin administration. In previous studies, exposure to triazine herbicides such as atrazine, terbuthylazine, and ametryn would cause oxidative stress, change antioxidant systems, and lead to DNA damage [39–41]. Thus, the increased bioavailability may pose a higher potential risk to health hazards when people are exposed to herbicides at a relatively high level after taking antibiotics. Then, we found that bioavailability was significantly increased under both 20 mg/kg and 2 mg/kg body weight of triazine herbicide exposure in the rats treated with antibiotic cocktails for 14 days. The result suggested that broad-spectrum antibiotic treatment over a long duration period may increase pesticide bioavailability in the host, thus leading to a greater health threat.

Antibiotic intake regardless of ampicillin or antibiotic cocktails could not only significantly reduce microbial amount (Fig. 3), but also alter microbial diversity and composition (Additional file 1: Figure S2, Figure S3 and Figure S6). After ampicillin or antibiotic cocktail treatment, the relative abundance of species from the genus *Ruminococcaceae* was decreased and species from the genus *Bacteroides* was increased. In previous studies, some bacteria such as *Pseudaminobacter*, *Pseudomonas*, and *Betaproteobacteria* from soil and groundwater were involved in the triazine herbicide degradation [42–44]. In this work, in vitro microbial metabolism assays showed that triazine herbicides could not be directly metabolized by gut microbiota from the antibiotic-treated or untreated rats, suggesting gut microbial metabolism may contribute little to the increased herbicide bioavailability. Meanwhile, the results from microsomal metabolism assays implied that antibiotics might not directly affect triazine herbicide metabolism by inhibiting the metabolic enzymes. Many previous studies indicated that gut microbiota could impact the metabolic physiology of hosts [45]. In the current study, it could be hypothesized that the antibiotic-changed microbiota may also affect the physiology of rats and finally lead to the increased herbicide bioavailability. Direct exposure to antibiotics produced complex intertwined effects including the changes of hepatic metabolic enzymes and gut microbiota, which made it difficult to confirm whether the increased bioavailability was related to gut microbiota.

Thus, we developed a microbial transplantation approach based on the antibiotic cocktail-treated rats, making gut microbiota the only variable that could directly evaluate the effect of gut microbiota on herbicide bioavailability. Fourteen-day antibiotic cocktail treatment greatly reduced gut bacterial amount, and the transplantation with normal microbiota made the microbial amount substantially rise (the normal microbiota rats) while the transplantation with antibiotic cocktail-treated microbiota almost kept the bacterial amount (the deficient microbiota rats). In other word, the microbial population of the normal microbiota rats was much larger than the deficient microbiota rats (Fig. 5a). After microbiota transplantation, the relative abundance of phylum *Firmicutes* and class *Coriobacteriia* showed a significantly increase in the normal microbiota rats. But in the case of the large difference in the amount of bacteria in the deficient microbiota rats and the normal microbiota rats, the effects of microbial diversity and composition on the increased herbicide bioavailability may be limited, so the altered microbial species in the deficient microbiota rats relative to the normal microbiota rats perhaps could not be considered as the specific bacteria contributing to the increased herbicide bioavailability. Oral bioavailability of triazine herbicides was significantly increased in the deficient microbiota rats, directly indicating that gut microbiota contributed to bioavailability.

However, how gut microbiota increased the herbicide bioavailability was not yet thoroughly understood. One important pathway may be that gut microbiota affects the metabolism of xenobiotics via regulating liver metabolic functions [46]. The triazine herbicide metabolism was mainly mediated by the CYP enzymes in phase I metabolism and glutathione S-transferase (GSH) in phase II metabolism according to previous studies [47–49], which was also proved in our microsomal metabolism assays added with enzymatic inhibitors (Additional file 1: Figure S13). We further examined differences in mRNA expression of these enzymes in the livers of the deficient microbiota and normal microbiota rats. The mRNA expression of enzymes CYP1A2, CYP2C11, CYP2D2, CYP2E1, CYP3A2, CYP3A11, GSTYc2, and GSTM2 was much lower in the deficient microbiota group, which was similar with previous findings. Selwyn et al. found that mRNA expression of Cyp3a family members Cyp3a11 and Cyp3a44 was significantly reduced in the livers of 90-day-old male germ-free mice compared to conventional mice [50], in addition to similar results for expression of GSHs, including Gsta1, Gstp1, Gstp2, and Gstm3 [51]. Kuno et al. also found that Cyp2b10, Cyp2c29, Cyp3a11, and Cyp4a12a expression was significantly lower in the liver cell membranes of germ-free mice than those of SPF mice [52]. Our results indicated that gut microbiota may reduce the efficiency of hepatic detoxification by regulating the expression of metabolic enzymes, thus resulting in an increase in the triazine herbicide bioavailability.

Absorption processes in the gastrointestinal tract are also factors that affect the xenobiotic bioavailability. Xenobiotics are mainly absorbed through the small intestine and circulated in the blood. There are many transport-related proteins that are mainly involved in epithelial cell absorption on the small intestinal mucosa surface. We studied the protein expression in the intestinal mucosa of the deficient microbiota and normal microbiota rats by label-free quantitative proteomics. Among the altered proteins, expression of Slc3a1 protein (accession number Q64319) was significantly increased in the deficient microbiota rats. Slc3a1 is a kind of transport protein and a member of the solute carrier (SLC) family. These proteins are involved in the transport, absorption, and distribution of xenobiotics like ibuprofen, methotrexate, and so on [53]. The expression of Slc3a1 protein significantly increased in the deficient microbiota rats, but there was no significant change in other proteins that played a major role in drug transport, including the multidrug resistance-associated protein 2 (ABCC2) and P-glycoprotein of ATP-binding cassette transporter (ABC) family and the organic anion transporter 2 of the SLC family proteins. Thus, small intestinal uptake may not be the main cause of the increased triazine herbicide levels.

## Conclusions

Here, we assessed the effects of antibiotics on pesticide bioavailability in rats and investigated the underlying mechanisms. The bioavailability of orally administered triazine herbicides was increased in rat treated with a single antibiotic (ampicillin) or an antibiotic cocktail. To assess whether gut microbiota affected pesticide bioavailability, we established a microbiota transfer model to directly prove that gut microbiota deficiency increased bioavailability via altering affected the mRNA expression of hepatic metabolic enzymes and small intestinal absorption-related proteins. Overall, the results indicated that antibiotics could enhance the bioavailability of triazine herbicides and thereby may increase the exposure risk. The results also suggested a new potential pathway where antibiotics affected the xenobiotic bioavailability and implied that gut microbiota could affect the fate of xenobiotics in the host. Abuse of antibiotics may increase the risks associated with xenobiotics, such as pesticides, and amplify their consequent toxic effects.

## Methods

### Animals

Animal experiments were approved and performed in accordance with the guidelines of the Institutional Animal Care and Use Committee of China Agricultural University (approval No. CAU20161122-4). Six-week-old male Sprague-Dawley rats were purchased from Beijing Vital River Laboratory Animal Technology Co., Ltd. (Beijing, China) and housed at 22 ± 2 °C in a standard cage in a specific pathogen-free facility with a 12:12-h light: dark cycle. Water and chow were available ad libitum. Ampicillin was dissolved in ultrapure water, and it was administered by gavage to the rats every day at 8:00, 16:00, and 23:00, with 90 mg/kg (body weight) in each dose. For the antibiotic cocktail treatment model, an antibiotic cocktail was dissolved in ultrapure water and administered by gavage to the rats once daily for 14 continuous days. A mixture of triazine herbicide standards was dissolved in corn oil containing 10% DMSO and the rats were fasted for 12 h before the herbicides were intragastrically administered. The blood samples were collected via the jugular vein with EDTA-containing tubes at 5, 10, 20, and 40 min and 1, 2, 3, 5, 8, 12, and 24 h after the herbicide administration and then were stored at − 20 °C for future use.

### Antibiotic dose

The recommended ampicillin dosage is 1–3 g/day for adult humans (medication guides from Chongqing Kerui pharmaceutical Co., Ltd., China). After converting the human dose to the animal equivalent dose based on the body surface area (Additional file 1: Table S5), the equivalent ampicillin dose for rats was 90–270 mg/kg/day (body weight). Thus, the rats were treated with 90 mg/kg body weight of ampicillin three times per day. According to previous research [37], the rats were given ampicillin, neomycin, gentamicin, and metronidazole (each at 1.75 mg/day) and vancomycin (at 0.875 mg/day) to establish an antibiotic-treated model. In summary, the maximum oral dose of these antibiotics was 7 mg/kg when calculated for an average rat weight of 250 g. Thus, the concentration of each antibiotic in blood circulation was not more than 7 mg/L, which was used in the microsomal assays.

### Analysis of triazine herbicide levels in blood

An aliquot of 200 μL of whole blood was added to 1 mL of acetonitrile and 200 μL of saturated NaCl solution, and the mixture was vortexed for 3 min, then centrifuged at 10,000 *g* for 1 min. The supernatant was dried at 35 °C under a nitrogen atmosphere and adjusted to a final volume with 100 μL of acetonitrile containing 50 μg/L of metamitron (internal standard) to obtain a solution for HPLC-MS/MS detection. Triazine herbicide levels were quantified by HPLC-MS/MS with a C_18_ columns (150 × 2.1 mm, 3 μm, Waters, Shanghai, China). Seventy percent acetonitrile and 30 % water (containing 0.1% formic acid) were used as the mobile phase, and the flow rate was 0.3 mL/min. The injection volume was 10 μL. The setup for the mass spectrometer were as follows: spray voltage of positive ionization mode 3000 V, vaporizer temperature 200 °C, sheath gas pressure 40 Arb, aux gas pressure 10 Arb; ion sweep gas pressure 0 Arb; collision gas pressure 1.5 mTorr. The specific selective reaction monitoring (SRM) was used and parameters of molecular fragmentation was presented in Additional file 1: Table S6. Method validation procedures and quality control data were showed in the Additional file 1: Table S7 and Table S8.

### Microsomal metabolism assay

Liver microsomes were prepared based on previously described methods [54, 55]. In brief, the rat liver was quickly removed after euthanasia and washed with 1.15% KCl solution to remove blood. After homogenized in an ice-cold SET solution (1 mM EDTA and 50 mM Tris-HCl, pH 7.4), the liver homogenate was centrifuged at 9000 *g* for 20 min, and the supernatants were subjected to two consecutive centrifugations at 100,000 *g* for 60 min. The microsomal pellet was resuspended in 50 mM Tris-HCl buffer containing 20% glycerol and stored at − 80 °C until use. In microsomal metabolism assay, the microsomes were diluted with Tris-HCl buffer (50 mmol/L, pH 7.4) and placed in a 37 °C water bath for pre-incubation for 5 min. An aliquot of 5 μL of triazine herbicides dissolved in ethanol and 5 μL of antibiotic cocktails dissolved in Tris-HCl buffer (gentamicin, neomycin, ampicillin, metronidazole, and vancomycin) were added to 440 μL of microsome incubation solutions and made that the final concentration of herbicides and antibiotics was respectively 50 μg/L (per herbicide) and 7 mg/L (per antibiotic). Then, 50 μL of NADPH (final concentration, 10 mmol/L) was added to initiate the reactions and the total incubation volume was 500 μL. Acetonitrile was added to stop the reactions after incubation at 0, 10, 20, 40, and 60 min. The control group was added with equivalent triazine herbicides without antibiotics. The extraction and detection were similar with the processes for blood and presented in Additional files.

### Microbial metabolism of triazine herbicides in vitro

The cecal feces samples were collected from the rats exposed to 3-day and 7-day ampicillin and 14-day antibiotic cocktails as well as from the corresponding control antibiotic-untreated rats. According to previous reports, the fecal samples were weighed and homogenized adequately with sterile physiological saline at a ratio of 1:4 (m/v). Then, the bacterial suspension was separated after centrifugation at 2000 g for 10 min. The general anaerobic medium (GAM) containing 0.3 g of L-cysteine hydrochloride, 0.3 g of sodium thioglycolate, 1.2 g of beef liver extract powder, 2.2 g of beef extract, 2.5 g of KH_2_PO_4_, 3 g of glucose, 3 g of soya peptone, 3 g of NaCl, 5 g of soluble starch, 5 g of yeast extract, 10 g of tryptone, 10 g of proteose peptone, 13.5 g of digestible serum powder, and 1000 mL of distilled water was used for the bacterial culture [56]. Ten microliters of bacterial suspension was inoculated into 990 μL of GAM containing the mixture of triazine herbicides at final concentration of 2 mg/L or 0.02 mg/L, and then incubated under anaerobic condition using a disposable O_2_-absorbing and CO_2_-generating agent (AnaeroPack Helico, Mitsubishi Gas Chemical Co., Inc., Tokyo, Japan) at 37 °C [57]. Degradation of herbicides in sterilized culture medium without bacteria was considered as negative control. All samples which had been incubated for 0, 1, 3, 5, 7, 12, 24, and 48 h were extracted with acetonitrile (two volumes of the culture medium). The extraction and detection of triazine herbicides were similar with the processes for microsomal experiments. Each group has five parallels.

### Microbial community analysis

Fresh feces of rats was collected and quickly placed in liquid nitrogen and then stored at − 80 °C. DNA was extracted from 0.25 g of feces using the QIAamp DNA Stool Kit (Qiagen, Gaithersburg, MD, USA). Then, the DNA samples were diluted and amplified by qPCR (Bio-Rad CFX96, USA) using the universal primers U515F and U806R (U515F, 5′-GTGYCAGCMGCCGCGGTAA-3′; U806R, 5′-GGACTACHVGGGTWTCTAAT-3′) with TransStart Green qPCR SuperMix (Transgene, AQ101-03). The qPCR reaction procedure was 1 cycle of 5 min at 95 °C, followed by 1 min at 56 °C and 40 cycles of 1 min at 72 °C. Total bacterial abundance was assessed via the standard curve with plasmid DNA as template.

To assess the microbial community composition, the hypervariable V3-V4 regions of 16S rRNA genes were amplified by PCR (5 min at 95 °C, followed by 27 cycles of 30 s at 95 °C, 30 s at 55 °C, 45 s at 72 °C, and a final 10 min at 72 °C) using the primers 341F and 806R (341F, 5′-CCTAYGGGRBGCASCAG-3′; 806R, 5′-GGACTACNNGGGTATCTAAT-3′). The PCR products were then purified with an AxyPrep DNA Gel Extraction Kit (Axygen, Union City, CA, USA) and quantified using a QuantiFluor™-ST (Promega, Madison, WI, USA) kit. Purified amplicons were pooled for sequencing on an Illumina MiSeq platform according to the manufacturer’s instructions. QIIME (version 1.17) was used to process raw fastq files. High-quality reads were obtained after quality filtering and clustered into operational taxonomic units (OTUs) at the 97% similarity cutoff using UPARSE (version 7.1 http://drive5.com/uparse/).

### Microbiota transplantation

Gut microbiota was collected from the cecal contents of donor rats and then diluted 10 times with saline. The solution was centrifuged and the supernatant was collected. Microbiota was then transplanted to the antibiotic-treated rats by a 1-mL gavage of the supernatant. The microbiota-recipient rats were housed in an isolator under specific pathogen-free conditions.

### Hepatic metabolic enzyme mRNA expression

RNAs from liver tissues were extracted using the TRNzol Reagent (Trangen Biotechnology Co., Ltd., China) and was reverse transcribed into cDNA using FastQuant RT kits (Trangen Biotechnology Co., Ltd.), followed by qPCR using SuperReal PreMix Plus (Trangen Biotechnology Co., Ltd.). Each quantification was performed in triplicate. Primer oligonucleotide sequences were presented in Additional file 1: Table S9.

### Label-free proteomics of the small intestinal mucosa

A 10-cm-long mucosal sample was taken from the rat terminal ileum [58] and immediately placed in liquid nitrogen and stored at − 80 °C until further testing. An aliquot of 150 μL of lysate (40 mM Tris-HCl, 7 M urea, 2 M thiourea, 1% DTT, and 1 mM EDTA) and 1.5 μL of protease inhibitor were added to the mucosal sample and then mixed. The mixture was treated by ultrasound to shear the tissue and high-speed centrifugation at 14,000 rpm for 40 min at 4 °C. The supernatant was collected and the protein content was quantified using the Bradford method. After quantification, 100 μg of protein was taken from each sample and 50 mM ammonium bicarbonate was added to adjust the volume to maintain the urea concentration below 1 M. DTT (final concentration 10 mM) was added to the solution. The mixture was incubated at 56 °C for 1 h and then cooled to room temperature. Iodoacetamide (final concentration 55 mM) was added to the solution and incubated at room temperature without light for 40 min. After 2 μg of trypsin was added, the solution was incubated at 37 °C for 14–16 h and freeze-dried to a volume of 100 μL. Then, the proteins were separated by a nanoLC (AB Sciex eksigent 425, USA). Two buffers were used for these analyses: (A) 0.1% formic acid aqueous solution and (B) 0.1% formic acid acetonitrile solution. The samples were loaded using an autosampler to the trapping C_18_ column (0.10 × 20 mm, 3 μm), and then separated with the analytical C_18_ column (0.75 × 150 mm, 5 μm) at a flow rate of 300 nL/min. The elution gradient is presented in Additional file 1: Table S10. Finally, the samples were identified by a Q-Exactive mass spectrometer (Thermo Scientific, USA). Full-scan MS spectra (350–1750 m/z) were acquired at a resolution of 70,000 with an automatic gain control (AGC) target value of 3e6. The full-scan maximum injection time was 20 ms, and the dynamic exclusion was set to 12.0 s. The dd-SIM was acquired at a resolution of 17,500 with an AGC target value of 2e5. The isolation window was set to 2.0 m/z, and the maximum injection time was 80 ms.

Significant differences of protein between the deficient microbiota group and the normal microbiota group were identified following three criteria: (1) contain at least two peptide segments with confidence coefficient greater than 95%; (2) with a minimum fold change (FC) of 2, FC ≥ 2 was upregulation and FC ≤ 0.5 was downregulation; (3) *P* < 0.05 in the independent sample *t*-test.

### Data analysis

The area under concentration-time curves (AUCs) were estimated using Origin 9.4 software (OriginLab, USA). Data were expressed as mean ± SD. Statistical analysis in comparison between two groups was performed using independent sample *t*-test and one-way ANOVA was used for comparison among multiple groups (SPSS 23.0 statistical software, USA). All results were considered statistically significant at *P* < 0.05.

## Additional files

Additional file 1:  Additional Figures, tables and methods (http://www.pantherdb.org/). (https://www.lascn.com/Item/506.aspx). (DOCX 1963 kb)

Additional file 2: Operational taxonomic units (OTUs) and taxonomy of fecal microbiota. (XLSX 113 kb)
