# Supplementary material for: Antibiotics may increase triazine herbicide exposure risk via disturbing gut microbiota
Source: Microbiome. 2018 Dec 13;6:224. doi: 10.1186/s40168-018-0602-5 (PMC6291969; doi:10.1186/s40168-018-0602-5)
Supplement: Supplementary file 1 — Additional Figures, tables and methods (http://www.pantherdb.org/). (https://www.lascn.com/Item/506.aspx). (DOCX 1963 kb) [file 40168_2018_602_MOESM1_ESM.docx]

**Supplementary Material**

Title: Antibiotics may increase triazine herbicide exposure risk via disturbing gut microbiota

Jing Zhan, Yiran Liang, Donghui Liu, Xiaoran Ma, Peize Li, Chang Liu, Xueke Liu, Peng Wang*, Zhiqiang Zhou*

Beijing Advanced Innovation Center for Food Nutrition and Human Health, College of Science, China Agricultural University, No. 2, West Yuanmingyuan Road, Beijing 100193, P.R. China

*Corresponding author:

Peng Wang, Tel: +86-010-62732937; Email: [wangpeng@cau.edu.cn](mailto:wangpeng@cau.edu.cn);

Zhiqiang Zhou, Tel: +86-10-62733547; Email: [zqzhou@cau.edu.cn](mailto:zqzhou@cau.edu.cn);

Beijing Advanced Innovation Center for Food Nutrition and Human Health, College of Science, China Agricultural University, No. 2, West Yuanmingyuan Road, Beijing 100193, P.R. China.

**Supplementary Figures**


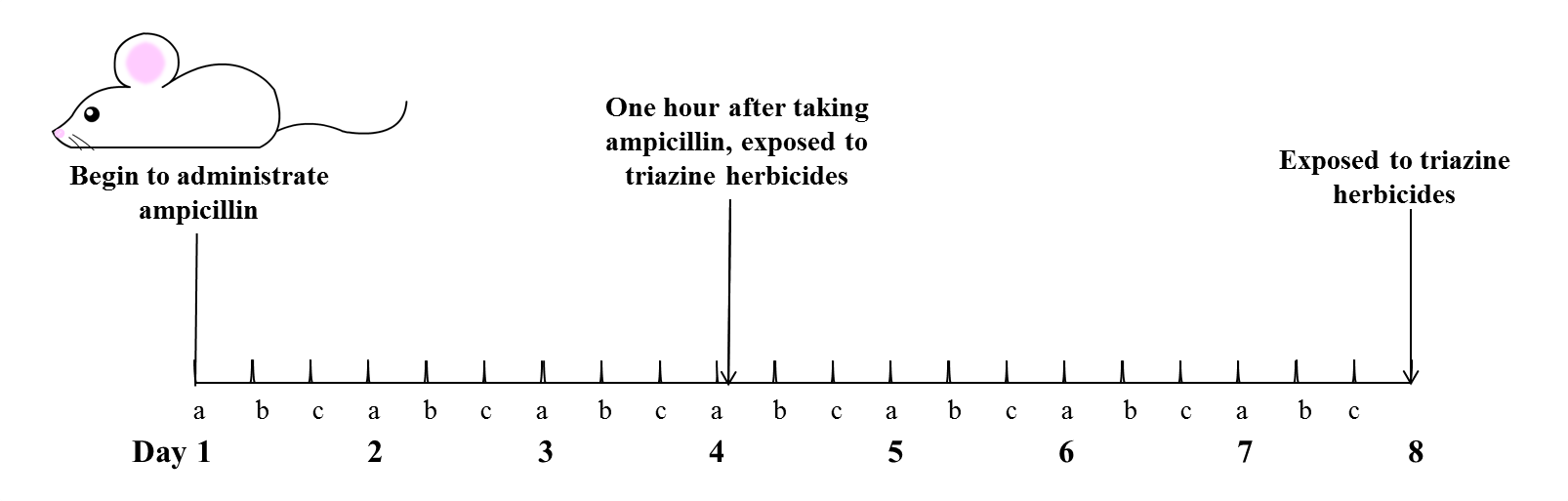


**Fig. S1** Experimental design of ampicillin exposure. Ampicillin was administered three times per day, and letter a, b and c means at 8:00, 16:00 and 23:00, respectively. Triazine herbicides (2 mg/kg body weight) were orally administered on the 4th and 8th day, respectively (n =5).


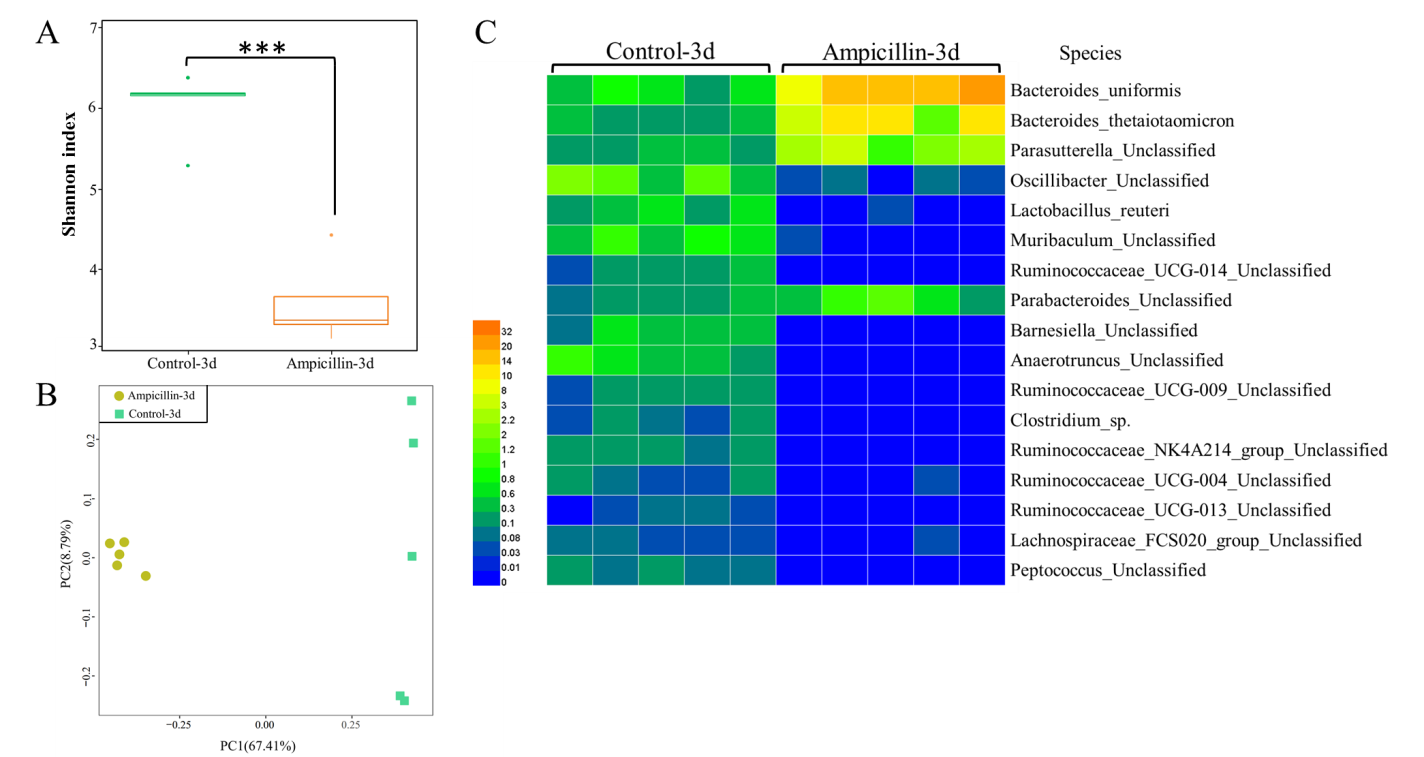


**Fig. S2** Effects of 3-day ampicillin treatment on the microbial diversity and composition (n=5). A: Shannon index for microbial diversity (independent sample t-test, ****P* < 0.001); B: Principal coordinate analyses (PCoA) of the Bray-Curtis comparison showing microbial composition dissimilarity between 3-day ampicillin treated and untreated rats’ feces; C: Heat map based on significantly changed microbial species in 3-day ampicillin rats relative to ampicillin untreated rats (the value of different color represented the relative abundance of species, independent sample t-test, *P* < 0.05 was considered as significantly difference).


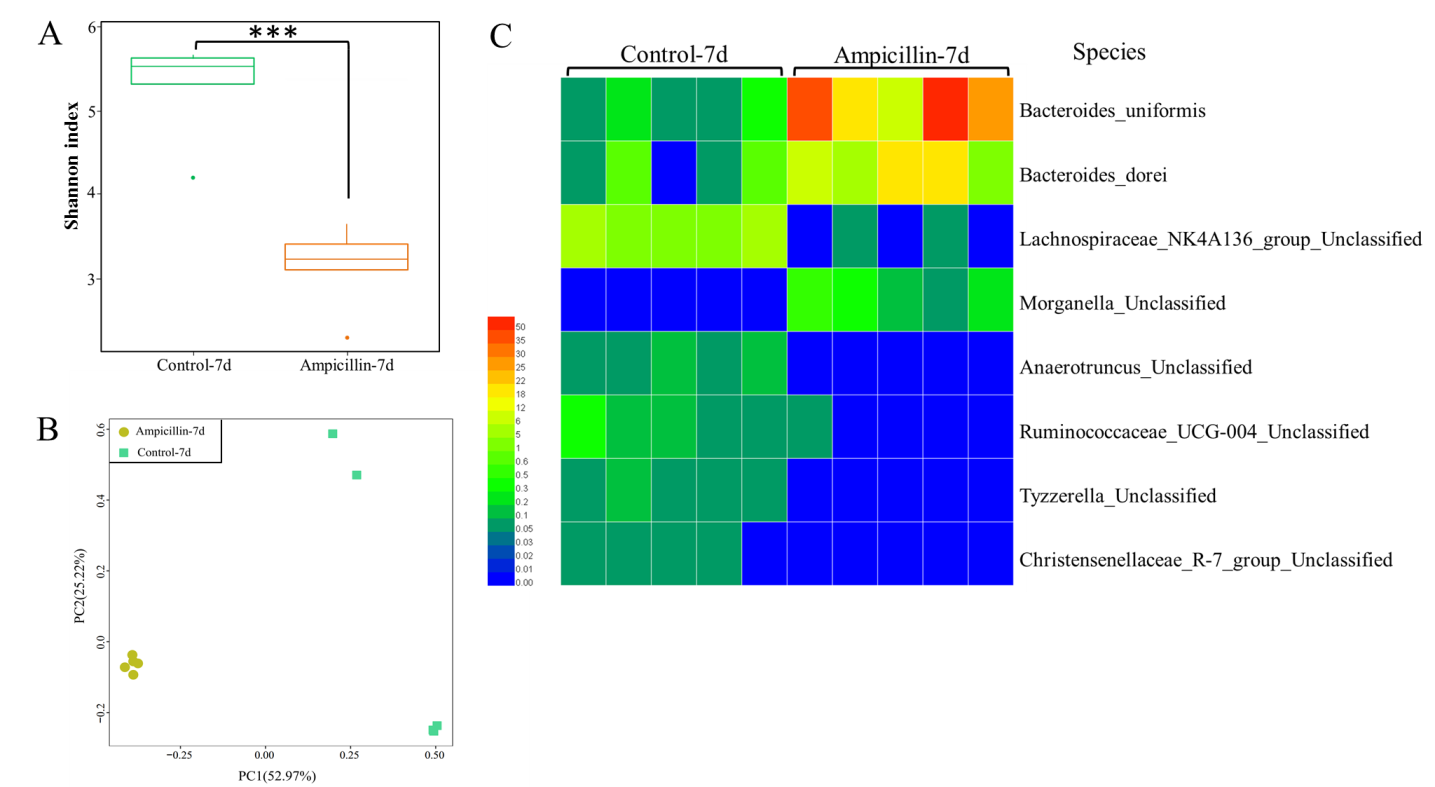


**Fig. S3** Effects of 7-day ampicillin treatment on the microbial diversity and composition (n=5). A: Shannon index for microbial diversity (independent sample t-test, ****P* < 0.001); B: Principal coordinate analyses (PCoA) of the Bray-Curtis comparison showing microbial composition dissimilarity between 7-day ampicillin treated and untreated rats’ feces; C: Heat map based on significantly changed microbial species in 7-day ampicillin rats relative to ampicillin untreated rats (the value of different color represented the relative abundance of species, independent sample t-test, *P* < 0.05 was considered as significantly difference).


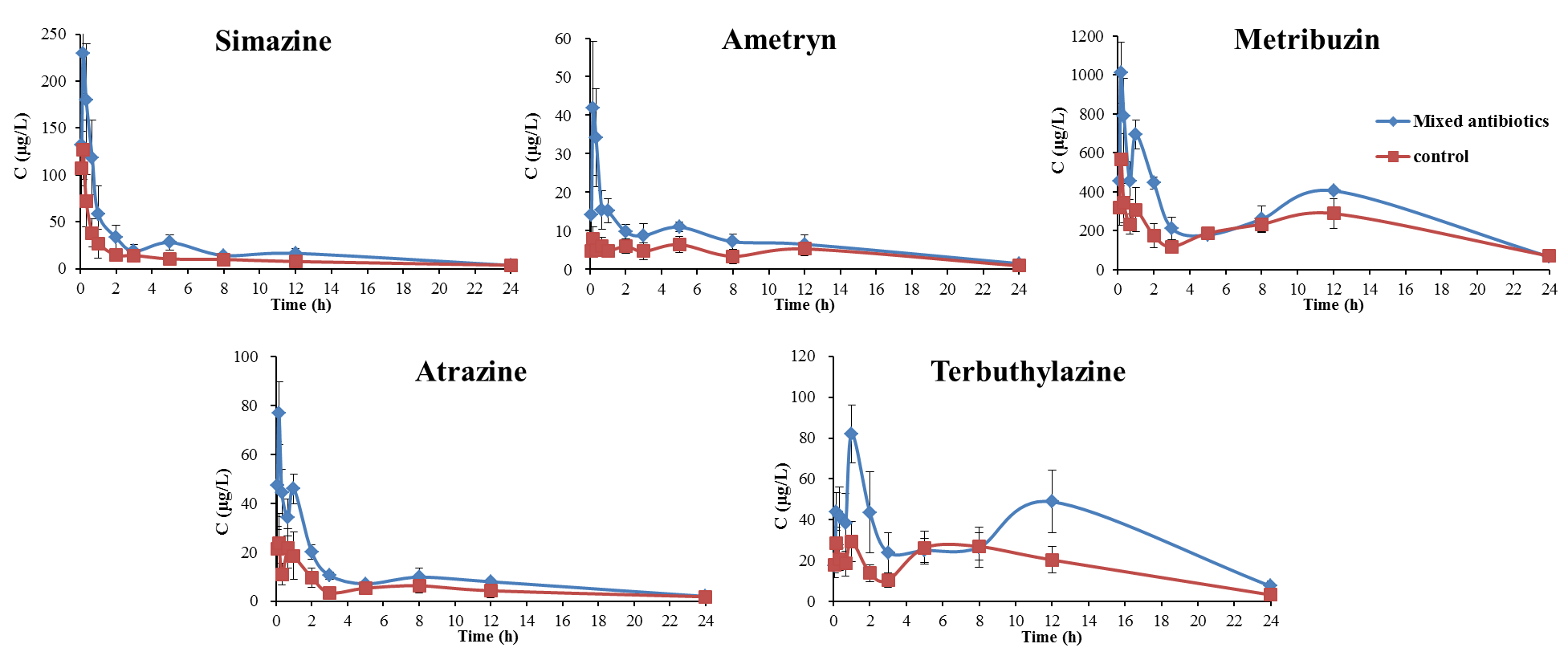


**Fig. S4** Concentration-time curve of triazine herbicides in blood of the rats exposed to 14-day antibiotic cocktails. Control rats were treated with equal volumes of water free from antibiotics. (20 mg/kg body weight of herbicides was administered on day 15, n =5).


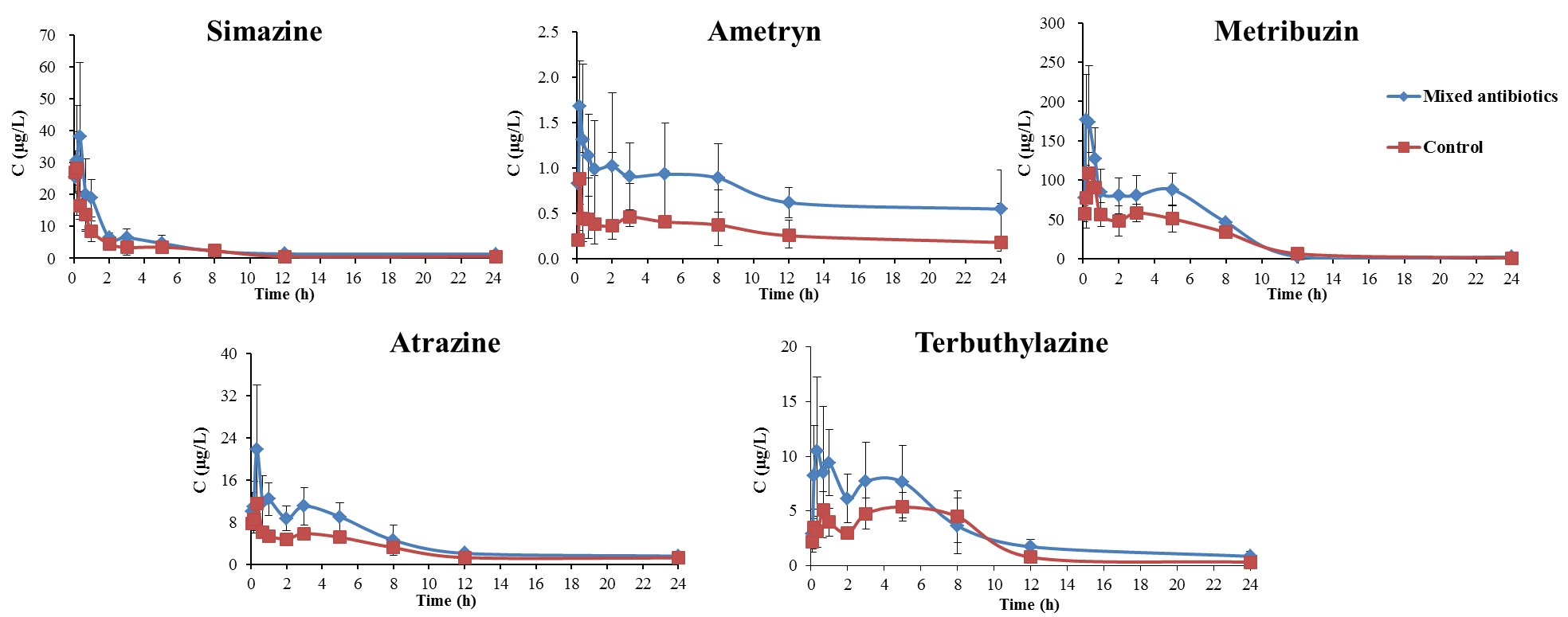


**Fig. S5** Concentration-time curve of triazine herbicides in blood of the rats exposed to 14-day antibiotic cocktails. Control rats were treated with equal volumes of water free from antibiotics. (2 mg/kg body weight of herbicides was administered on day 15, n =5).


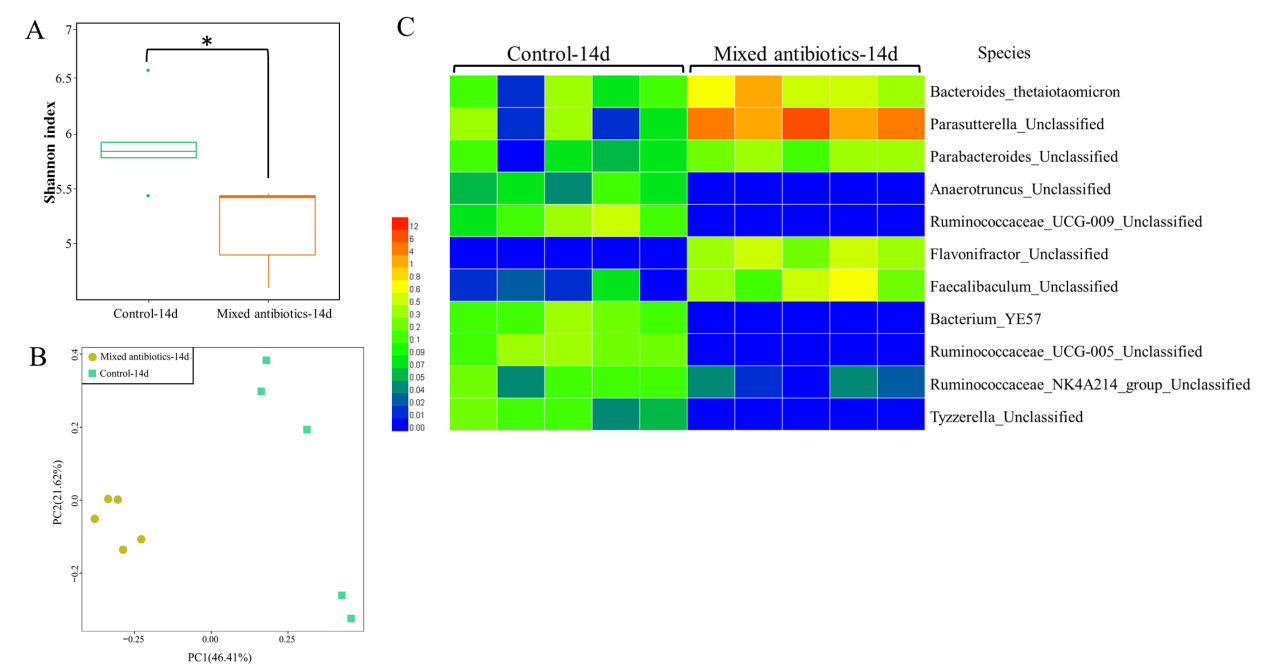


**Fig. S6** Effects of 14-day antibiotic cocktail treatment on the microbial diversity and composition (n=5). A: Shannon index for microbial diversity (independent sample t-test, **P* < 0.05); B: Principal coordinate analyses (PCoA) of the Bray-Curtis comparison showing microbial composition dissimilarity between 14-day antibiotic cocktail treated and untreated rats’ feces; C: Heat map based on significantly changed microbial species in 14-day antibiotic cocktail treated rats relative to antibiotic untreated rats (the value of different color represented the relative abundance of species, independent sample t-test, *P* < 0.05 was considered as significantly difference).


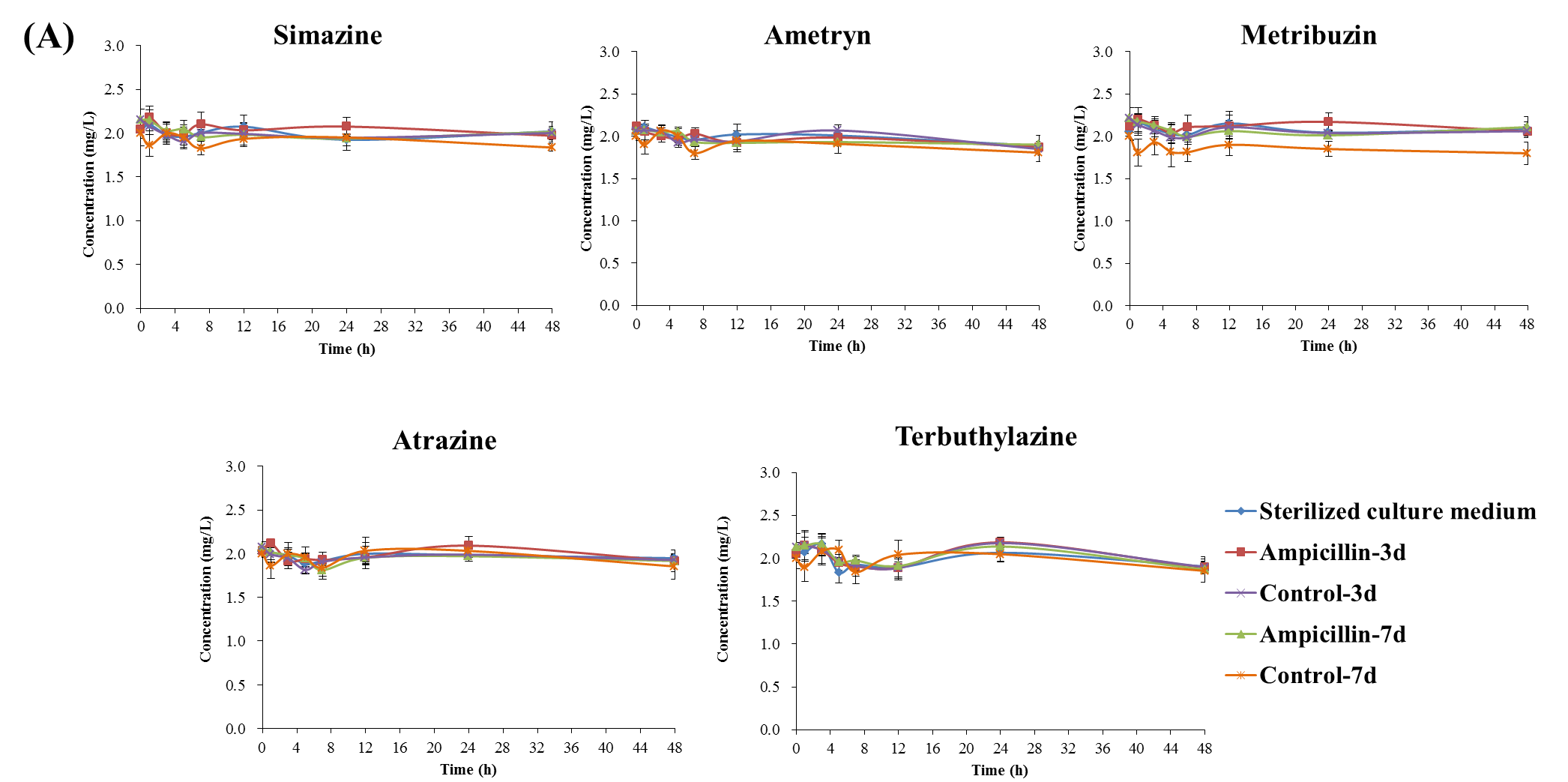


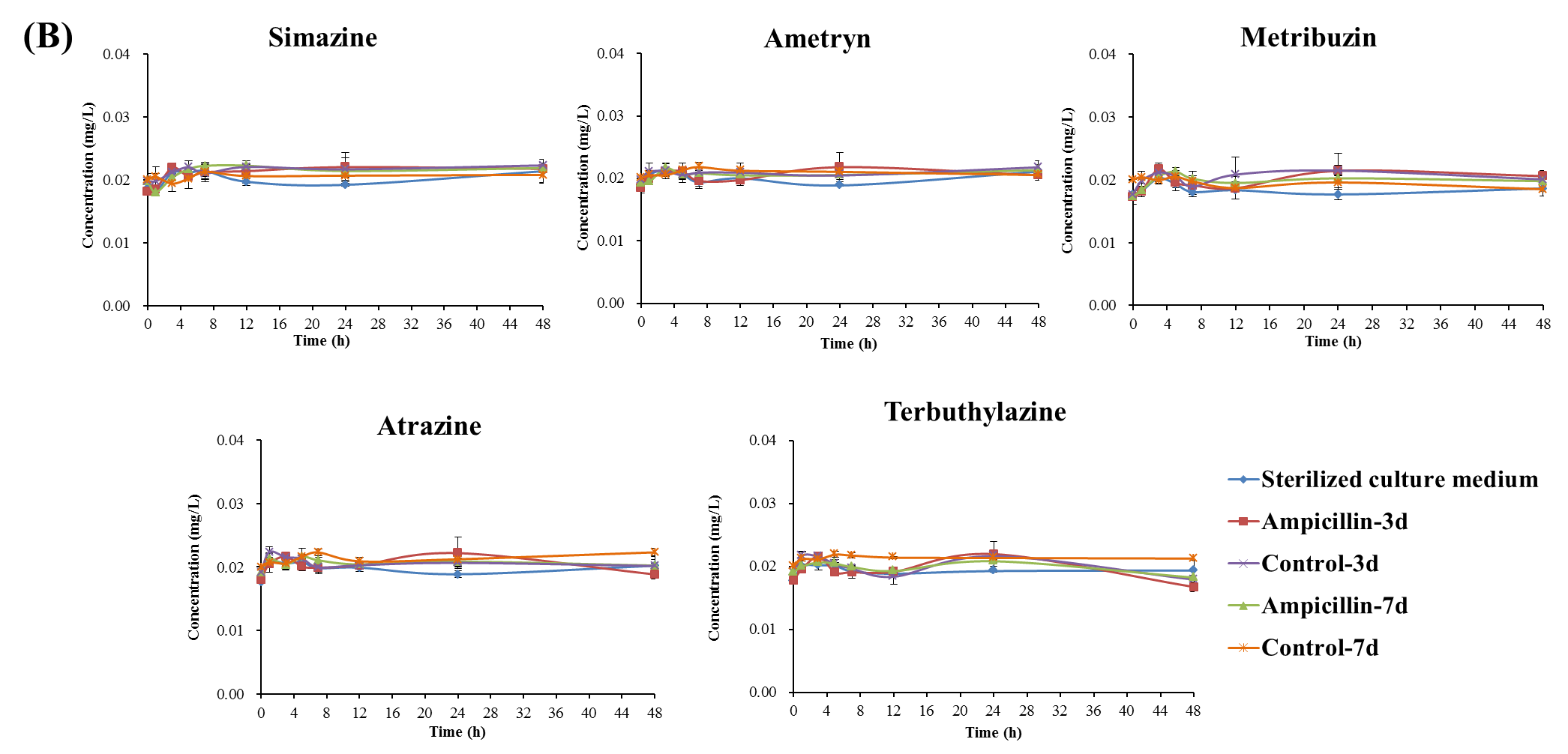


**Fig. S7** (A) Degradation of 2 mg/L of triazine herbicides by gut microbiota from 3-day and 7-day ampicillin-treated and control rats; (B) Degradation of 0.02 mg/L of triazine herbicides by gut microbiota from 3-day and 7-day ampicillin-treated and control rats (n=5).


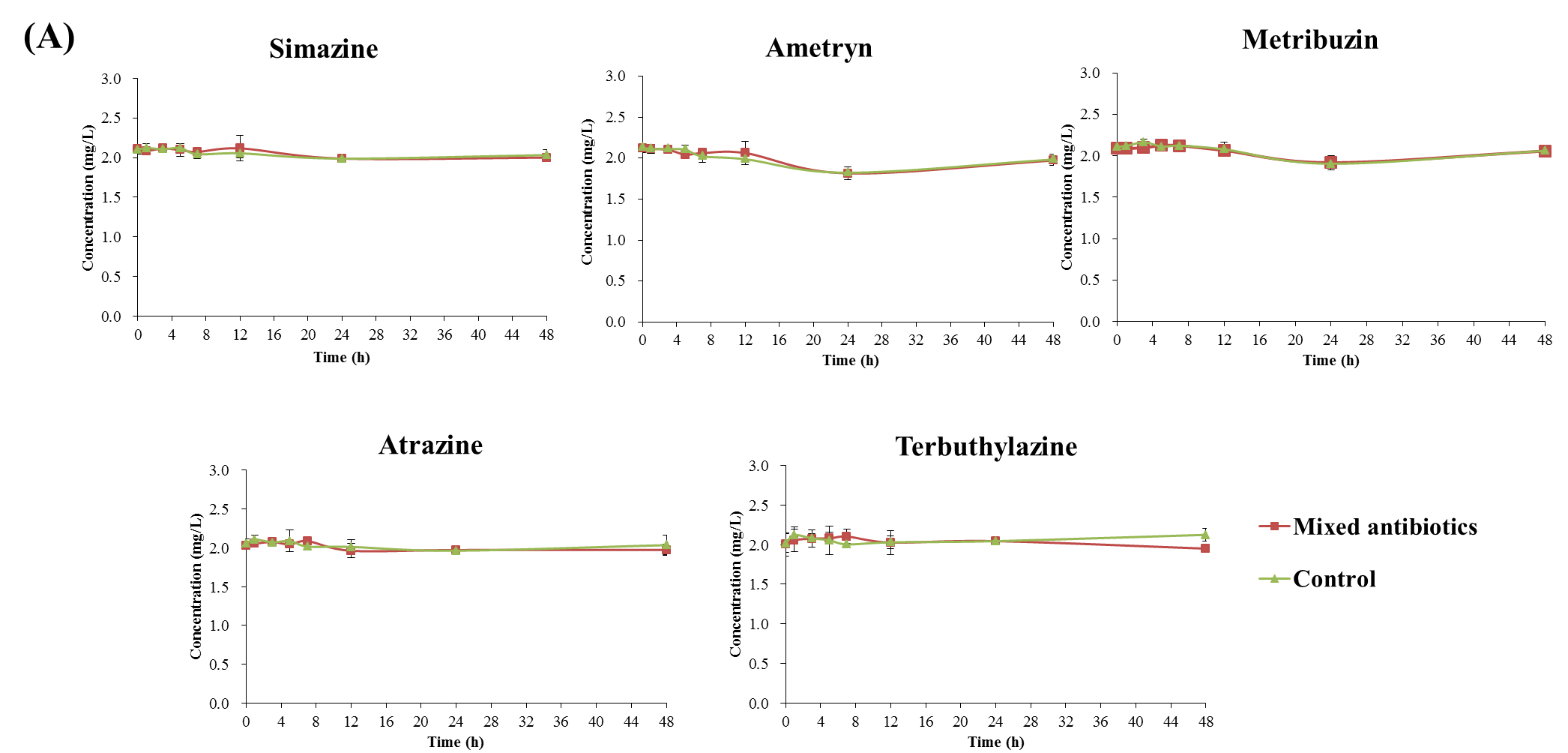


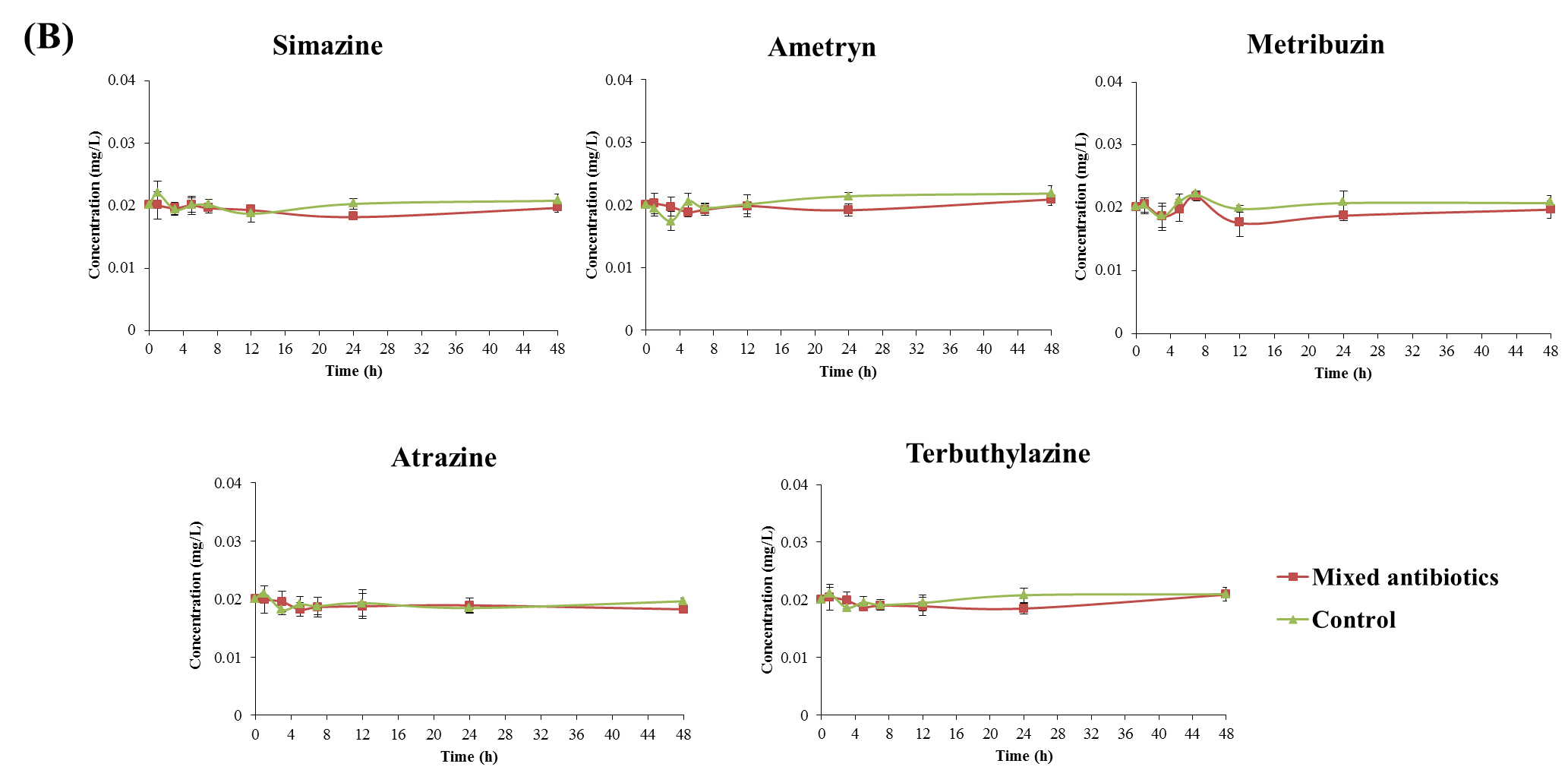


**Fig. S8** (A) Degradation of 2 mg/L of triazine herbicides by gut microbiota from 14-day antibiotic cocktail-treated and control rats; (B) Degradation of 0.02 mg/L of triazine herbicides by gut microbiota from 14-day antibiotic cocktail-treated and control rats (n=5).


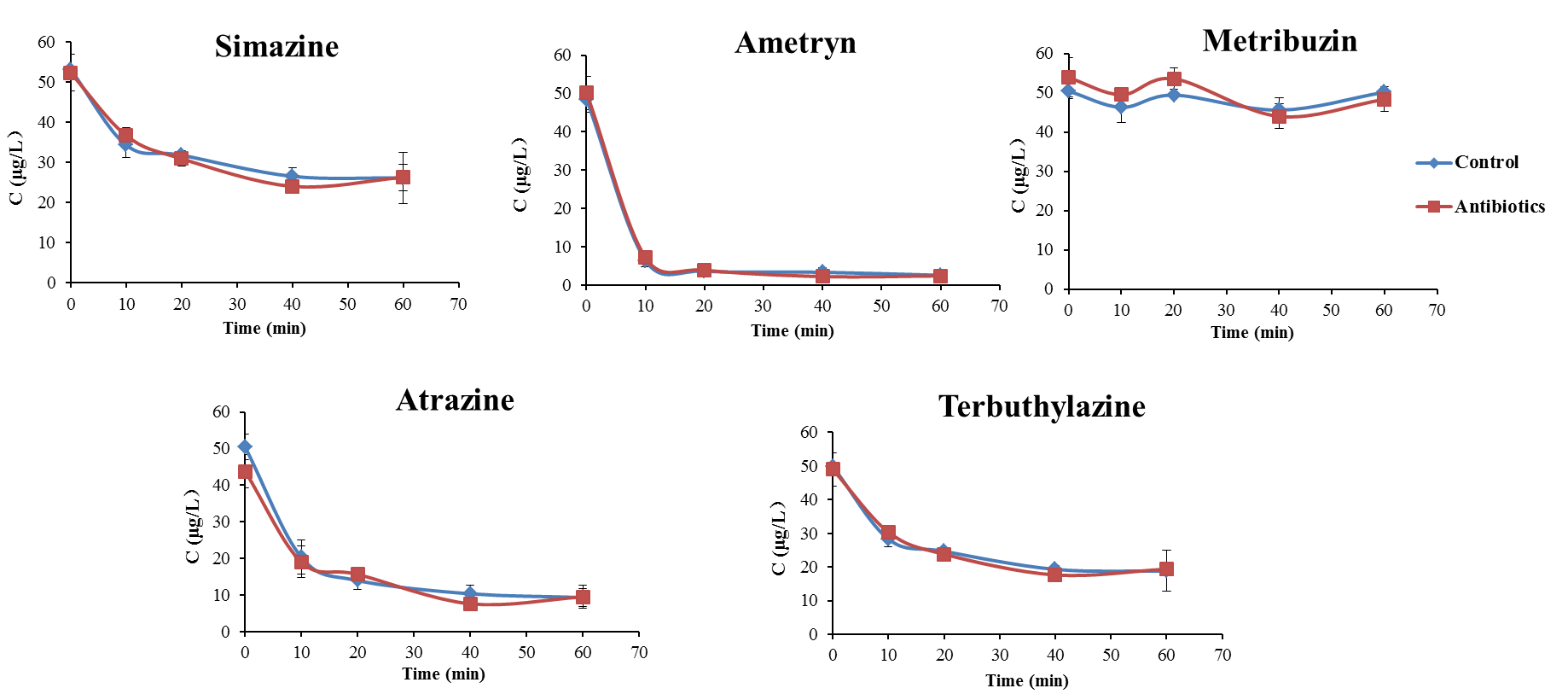


**Fig. S9** Effects of antibiotics on the metabolism of triazine herbicides in the liver microsomal assay (n=3).


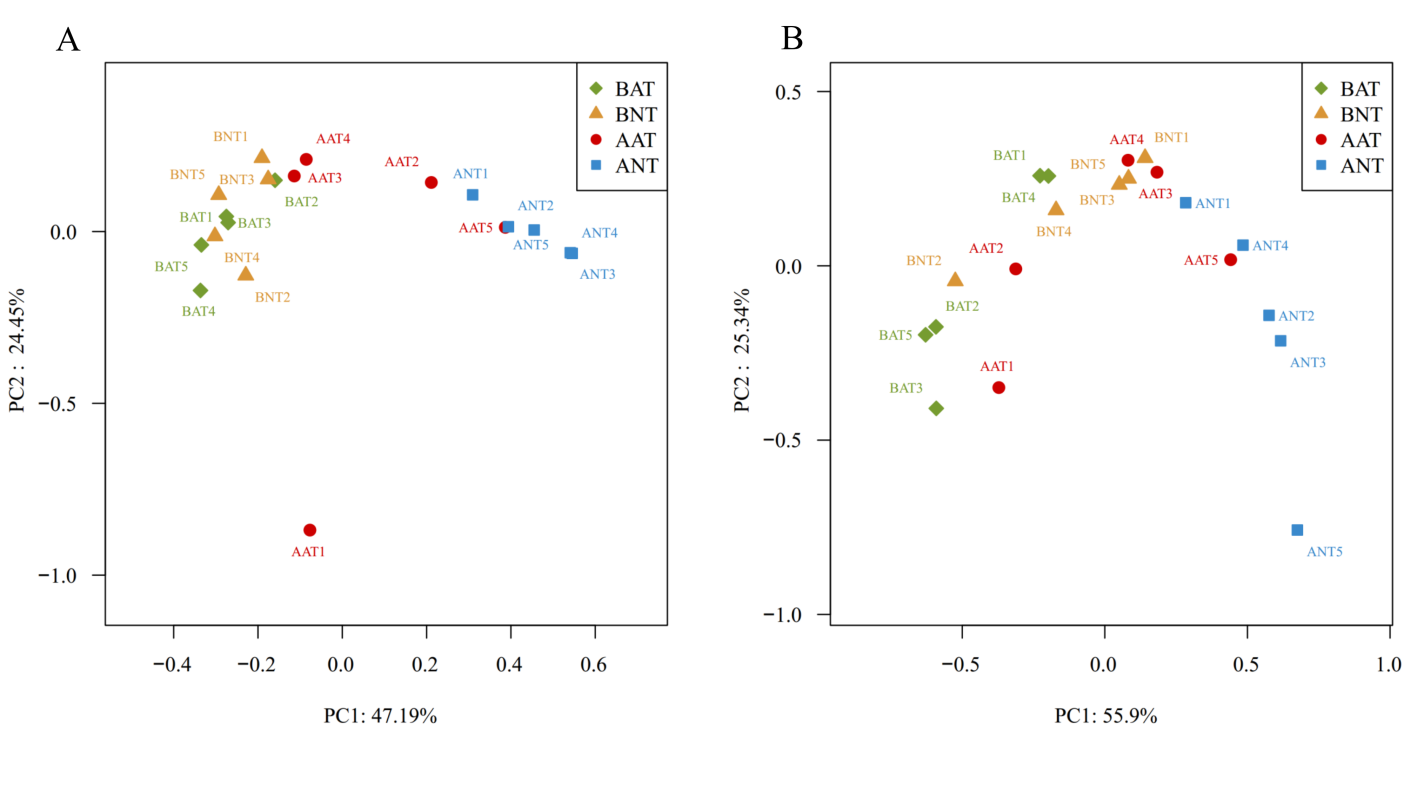


**Fig. S10** Principal coordinate analysis (PCoA) based on unweighted (A) and weighted (B) UniFrac comparison of gut microbiota in rats before and after microbiota transplantation (BAT: before antibiotic-treated microbiota transfer; BNT: before normal-treated microbiota transfer samples; AAT: after antibiotic-treated microbiota transfer; ANT: after normal-treated microbiota transfer, n=5).


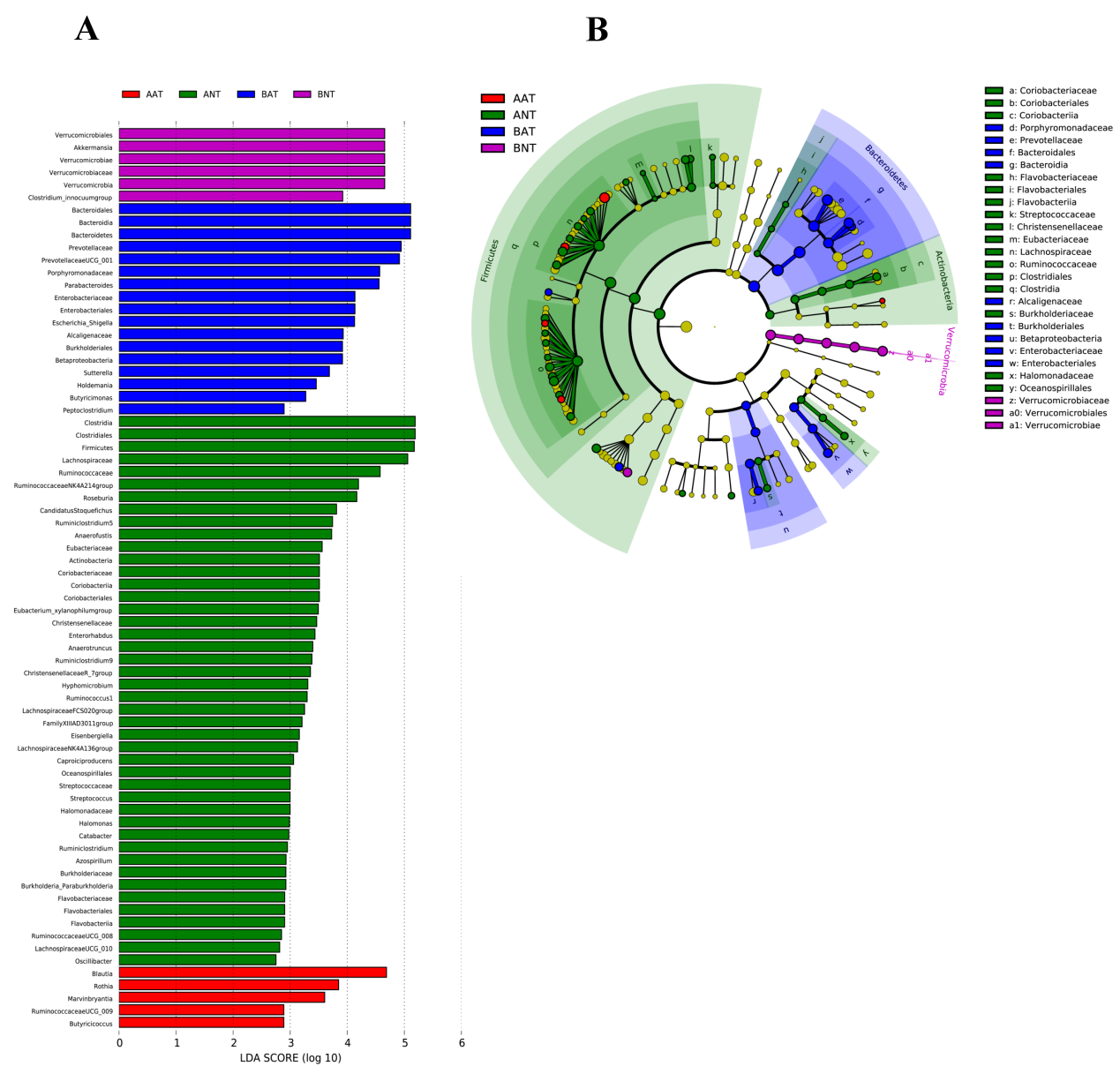


**Fig. S11** LEfSe analysis based on ANOVA followed by Bonferroni’s test of gut microbiota in rats before and after microbiota transplantation. A: LDA scores histogram showing the relative abundant of significantly different species among BAT, BNT, AAT and ANT samples (P < 0.05); B: Cladograms representing taxa enriched in BAT, BNT, AAT and ANT samples (BAT: before antibiotic-treated microbiota transfer; BNT: before normal-treated microbiota transfer samples; AAT: after antibiotic-treated microbiota transfer; ANT: after normal-treated microbiota transfer, n=5).


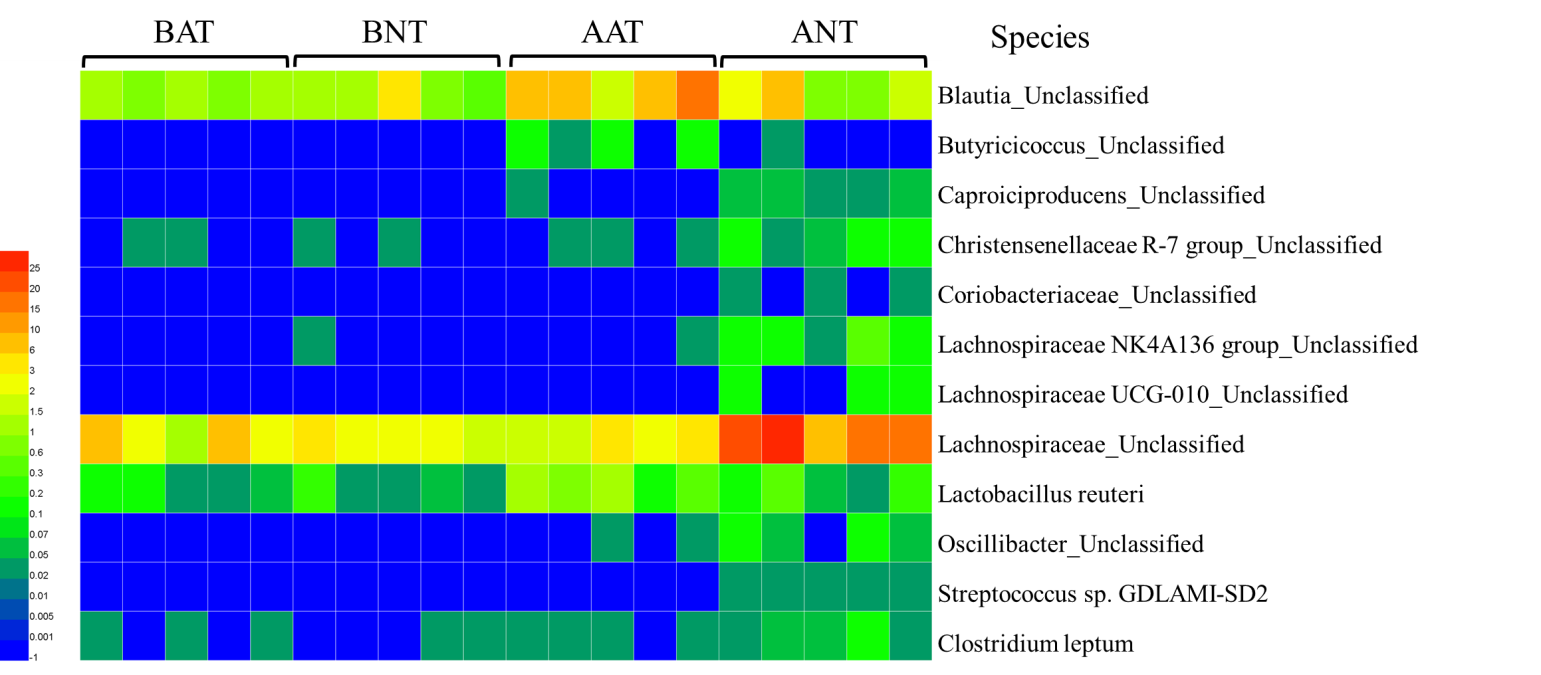


**Fig. S12** Heat map based on significantly changed microbial species in the gut microbiota of rats before and after microbiota transplantation (BAT: before antibiotic-treated microbiota transfer; BNT: before normal-treated microbiota transfer samples; AAT: after antibiotic-treated microbiota transfer; ANT: after normal-treated microbiota transfer; the value of different color represented the relative abundance of species, one-way ANOVA followed by Bonferroni’s test, *P* < 0.05 was considered as significantly difference, n=5).


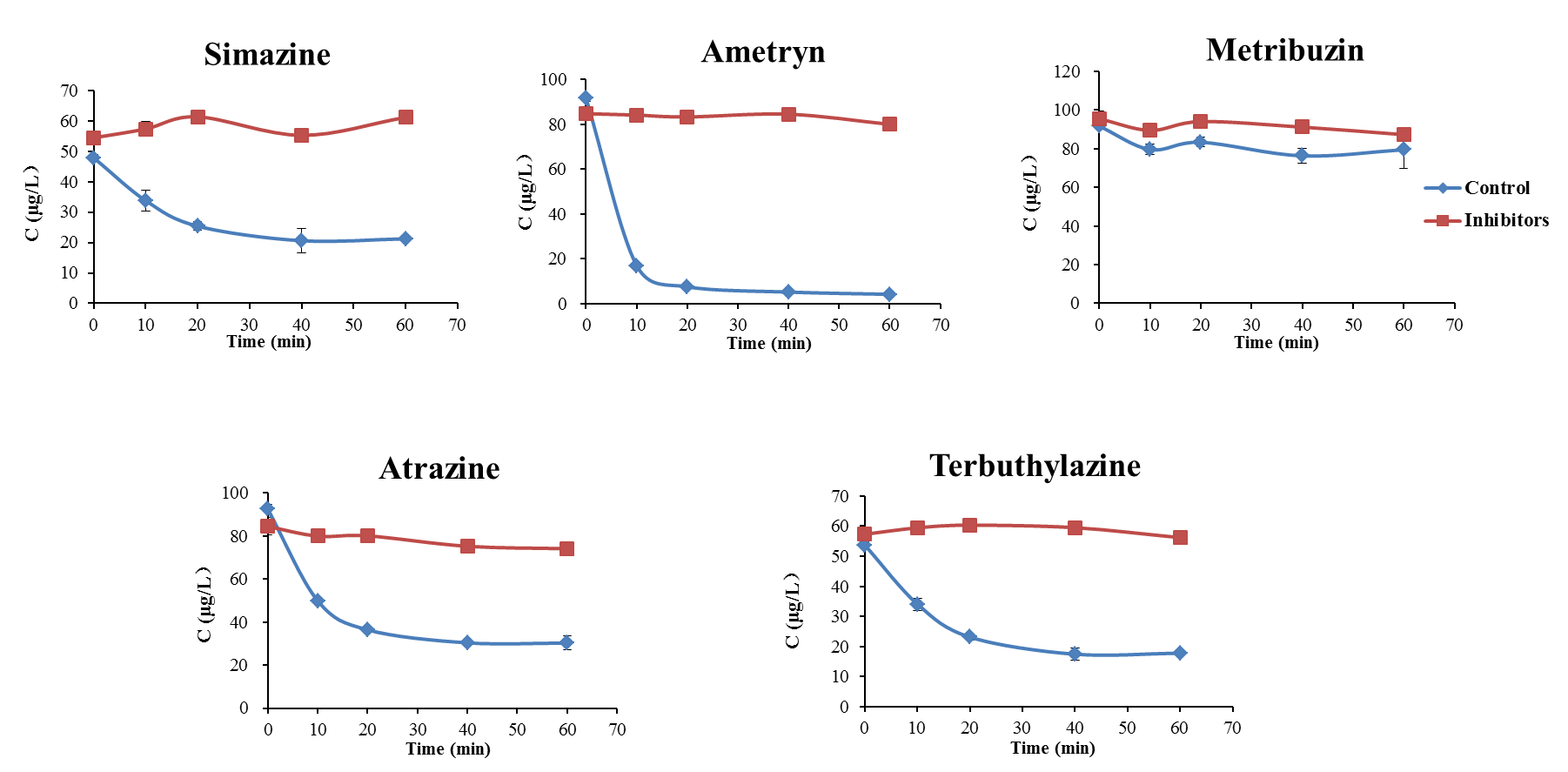


**Fig. S13** Effects of enzyme inhibitors on the metabolism of triazine herbicides in the liver microsomal assay. An aliquot of 5 μL of triazine herbicides (final concentration: 50 μg/L of simazine, metribuzin and terbuthylazine; 100 μg/L of ametryn and atrazine) and 5 μL of enzyme inhibitors (0.25 mM ketoconazole, cimetidine, propafenone and fluoxetine) were added to the microsome solutions. The control group was treated with triazine herbicides without enzyme inhibitors (n=3).


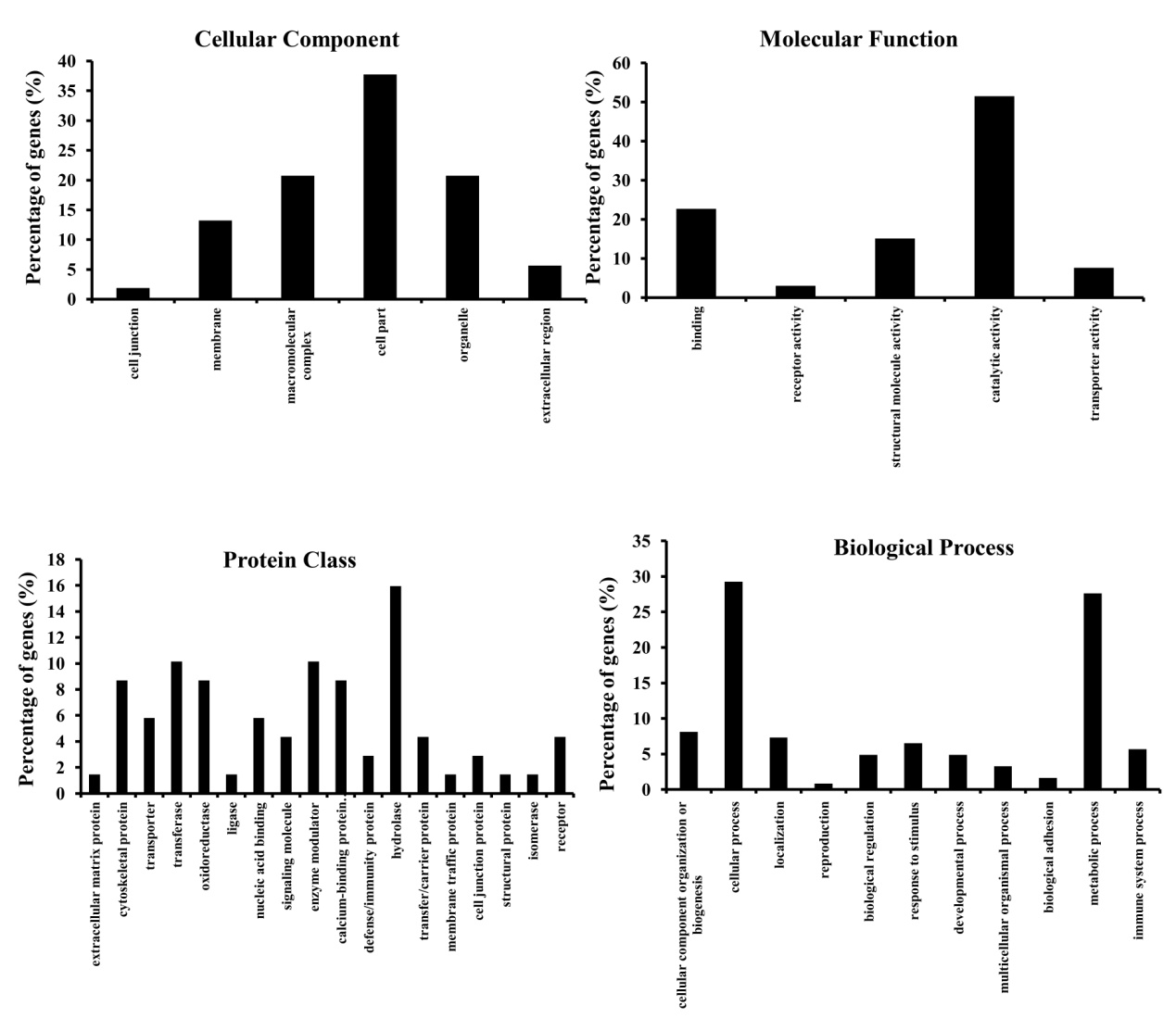


**Fig. S14** Classification analysis of the differently expressed proteins. A classification analysis of the differently expressed proteins was carried out in PANTHER (http://www.pantherdb.org/). From the perspective of cellular component, these protein were involved in cell part, organelle and macromolecular. From the perspective of molecular function, these protein were involved in catalytic activity, binding and structural molecule activity. From the perspective of protein class, these protein were involved in hydrolase and transferase. From the perspective of biological process, these protein were involved in metabolic process, cellular process and biological regulation (n=3).

**Supplementary Tables**

**Table S1** The AUC, C_max_ and increase rate of triazine herbicides exposure following 3-day and 7-day ampicillin treatments (the asterisk represented significant difference, *P* < 0.05, n=5).

|  | After 3-day ampicillin treatment | | | |  | After 7-day ampicillin treatment | | | |
| --- | --- | --- | --- | --- | --- | --- | --- | --- | --- |
| Herbicide | Treatment | AUC _0~24h_  (μg∙h/L) | C_max_  (μg/L) | Increase rate of AUC |  | Treatment | AUC _0~24h_  (μg∙h/L) | C_max_  (μg/L) | Increase rate of AUC |
| Simazine | Ampicillin | 44 | 77.9 | 3.31% |  | Ampicillin | 82.2 | 56.1 | 52.10% |
|  | Control | 42.6 | 70.3 |  |  | Control | 54 | 35.7 |  |
| Ametryn | Ampicillin | 12.0 * | 1.7 | 55.60% |  | Ampicillin | 25.0 * | 4.1 | 75.79% |
|  | Control | 7.7 | 1.6 |  |  | Control | 14.2 | 1.8 |  |
| Metribuzin | Ampicillin | 638.4 * | 200.9 | 29.08% |  | Ampicillin | 1780.7 * | 216.1 | 59.26% |
|  | Control | 494.6 | 175.6 |  |  | Control | 1118.1 | 215 |  |
| Atrazine | Ampicillin | 42.5 | 34.7 | 26.43% |  | Ampicillin | 139.1 | 28.8 | 55.74% |
|  | Control | 33.6 | 32.7 |  |  | Control | 89.3 | 19.3 |  |
| Terbuthylazine | Ampicillin | 58.8 * | 10.1 | 50.32% |  | Ampicillin | 145.7 * | 9.6 | 93.15% |
|  | Control | 39.1 | 9.5 |  |  | Control | 75.4 | 8.9 |  |

**Table S2** The AUC, C_max_ and increase rate of 20 mg/kg and 2 mg/kg (body weight) of triazine herbicides exposure following antibiotic cocktail treatment (the asterisk represented significant difference, *P* < 0.05, n=5).

| 20 mg/kg per herbicide | | | | |  |  | 2 mg/kg per herbicide | | | | |
| --- | --- | --- | --- | --- | --- | --- | --- | --- | --- | --- | --- |
| Herbicide | Treatment | AUC _0~24h_  (μg∙h/L) | C_max_  (μg/L) | Increase rate of AUC | |  | Treatment | AUC _0~24h_  (μg∙h/L) | C_max_  (μg/L) | Increase rate of AUC |  |
| Simazine | Antibitics | 495.1 * | 229.5 * | 94.92% | |  | Antibitics | 88* | 38.2* | 64.79% |  |
|  | Control | 254 | 127 |  |  |  | Control | 53.4 | 28.3 |  |  |
| Ametryn | Antibitics | 166.1 * | 41.8 * | 72.30% | |  | Antibitics | 17.6* | 1.7* | 151.43% |  |
|  | Control | 96.4 | 7.9 |  |  |  | Control | 7 | 0.9 |  |  |
| Metribuzin | Antibitics | 6695.3 * | 1012.0 * | 39.42% | |  | Antibitics | 784.3* | 176.7* | 44.78% |  |
|  | Control | 4802.1 | 564.8 |  |  |  | Control | 541.7 | 108.4 |  |  |
| Atrazine | Antibitics | 229.2 * | 76.9 * | 86.94% | |  | Antibitics | 111* | 21.8* | 66.67% |  |
|  | Control | 122.6 | 23.8 |  |  |  | Control | 66.6 | 11.6 |  |  |
| Terbuthylazine | Antibitics | 755.0 * | 82.0 * | 85.11% | |  | Antibitics | 81.5* | 10.5* | 53.77% |  |
|  | Control | 407.9 | 29.4 |  |  |  | Control | 53 | 5.1 |  |  |

**Table S3** The AUC, C_max_ and increase rate of 2 mg/kg (body weight) of triazine herbicides exposure in the deficient and normal microbiota rats (the asterisk represented significant difference, *P* < 0.05, n=5).

| Herbicide | Treatment | AUC _0~24h_  (μg∙h/L) | C_max_  (μg/L) | Increase rate of AUC |
| --- | --- | --- | --- | --- |
| Simazine | Deficient microbiota | 117* | 196.9* | 98.31% |
|  | Normal Microbiota | 59 | 31.3 |  |
| Ametryn | Deficient microbiota | 6.7* | 3.3* | 21.82% |
|  | Normal Microbiota | 5.5 | 0.9 |  |
| Metribuzin | Deficient microbiota | 1745.8* | 568* | 74.35% |
|  | Normal Microbiota | 1001.3 | 163.8 |  |
| Atrazine | Deficient microbiota | 109.3* | 100.7* | 78.01% |
|  | Normal Microbiota | 61.4 | 16.6 |  |
| Terbuthylazine | Deficient microbiota | 99.6* | 38.1* | 54.90% |
|  | Normal Microbiota | 64.3 | 10.5 |  |

**Table S4** Significantly different proteins and the fold change of deficient microbiota rats relative to the normal microbiota rats as well as *p*-value in the independent sample t-test (n=3).

| Accession number | | Gene symbols | Protein names | Fold change | p-value |
| --- | --- | --- | --- | --- | --- |
| **Up-regulated** | |  |  |  |  |
| G3V9U7 | Atp2a3 | Calcium-transporting ATPase | 37.15 | 0.01 |  |
| P31399 | Atp5h | ATP synthase subunitd, mitochondrial | 24.75 | 0.01 |  |
| D4AB20 | B3galt5 | Hexosyltransferase | 12.37 | 0.01 |  |
| P21571 | Atp5j | ATP synthase-coupling factor 6, mitochondrial | 12.07 | 0.02 |  |
| P21708 | Mapk3 | Mitogen-activated protein kinase 3 | 11.63 | 0.01 |  |
| G3V6P8 | Gng12 | Guanine nucleotide-binding protein subunit gamma | 11.52 | 0.01 |  |
| F1LS48 | Acat2 | Acetyl-CoA acetyltransferase, cytosolic | 9.20 | 0.02 |  |
| P01048 | Map1 | T-kininogen 1 | 9.16 | 0.02 |  |
| D3ZKB6 | Acat2l1 | Acetyl-CoA acetyltransferase 2-like 1 | 8.81 | 0.02 |  |
| P13803 | Etfa | Electron transfer flavoprotein subunit alpha, mitochondrial | 8.36 | 0.02 |  |
| D3ZE15 | LOC100911483 | NADH dehydrogenase [ubiquinone] 1 alpha subcomplex subunit 13-like | 8.26 | 0.01 |  |
| Q498D9 | Gipc2 | PDZ domain-containing protein GIPC2 | 8.02 | 0.02 |  |
| P62828 | Ran | GTP-binding nuclear protein Ran | 7.34 | 0.02 |  |
| Q6P9U0 | Serpinb6 | Serine (Or cysteine) peptidase inhibitor | 7.18 | 0.00 |  |
| P25113 | Pgam1 | Phosphoglycerate mutase 1 | 6.01 | 0.02 |  |
| F1M8E9 | Lyz2 | Lysozyme | 5.68 | 0.01 |  |
| D3ZZC1 | Txndc5 | RCG43947 | 5.67 | 0.02 |  |
| Q6IFU9 | Krt16 | Keratin 16 | 5.67 | 0.02 |  |
| Q4KMC4 | Gfpt2 | Glutamine--fructose-6-phosphate aminotransferase [isomerizing] 2 | 5.63 | 0.02 |  |
| Q9ET64 | Smpd2 | Sphingomyelin phosphodiesterase 2 | 5.58 | 0.01 |  |
| Q5U3Z7 | Shmt2 | Serine hydroxymethyltransferase | 5.52 | 0.01 |  |
| Q5RJR2 | Twf1 | Twinfilin-1 | 5.41 | 0.02 |  |
| P62268 | Rps23 | 40S ribosomal protein S23 | 5.38 | 0.01 |  |
| P35171 | Cox7a2 | Cytochrome c oxidase subunit 7A2, mitochondrial | 5.02 | 0.01 |  |
| B5DF46 | Pmm2 | Phosphomannomutase | 5.01 | 0.02 |  |
| Q9Z1L1 | Cldn7 | Claudin-7 | 4.82 | 0.01 |  |
| B2GV73 | Arpc3 | Actin-related protein 2/3 complex subunit 3 | 4.70 | 0.02 |  |
| Q68FY0 | Uqcrc1 | Cytochrome b-c1 complex subunit 1, mitochondrial | 4.27 | 0.02 |  |
| P62890 | Rpl30 | 60S ribosomal protein L30 | 4.21 | 0.02 |  |
| P62963 | Pfn1 | Profilin-1 | 4.13 | 0.02 |  |
| Q66HT1 | Aldob | Fructose-bisphosphate aldolase | 3.95 | 0.02 |  |
| P31977 | Ezr | Ezrin | 3.87 | 0.02 |  |
| P05065 | Aldoa | Fructose-bisphosphate aldolase A | 3.76 | 0.02 |  |
| F1LUM5 | Tubal3 | Tubulin alpha chain | 3.72 | 0.02 |  |
| Q6Q0N1 | Cndp2 | Cytosolic non-specific dipeptidase | 3.71 | 0.02 |  |
| P24051 | Rps27l | 40S ribosomal protein S27-like | 3.60 | 0.02 |  |
| P05943 | S100a10 | Protein S100-A10 | 3.52 | 0.02 |  |
| Q66HG5 | Tm9sf2 | Transmembrane 9 superfamily member 2 | 3.46 | 0.02 |  |
| D3ZD09 | Cox6b1 | Cytochrome c oxidase subunit | 3.46 | 0.01 |  |
| Q6AY09 | Hnrnph2 | Heterogeneous nuclear ribonucleoprotein H2 | 3.39 | 0.02 |  |
| F1LNF0 | Myh14 | Myosin heavy chain 14 | 3.32 | 0.02 |  |
| D4A4D5 | LOC498555 | Uncharacterized protein | 3.24 | 0.02 |  |
| Q5XHY5 | Tars | Threonine--tRNA ligase, cytoplasmic | 3.10 | 0.02 |  |
| P11951 | Cox6c2 | Cytochrome c oxidase subunit 6C-2 | 3.02 | 0.00 |  |
| Q5M9I5 | Uqcrh | Cytochrome b-c1 complex subunit 6, mitochondrial | 3.02 | 0.01 |  |
| P06685 | Atp1a1 | Sodium/potassium-transporting ATPase subunit alpha-1 | 3.02 | 0.02 |  |
| P24090 | Ahsg | Alpha-2-HS-glycoprotein | 2.86 | 0.00 |  |
| Q6B345 | S100a11 | Protein S100-A11 | 2.82 | 0.02 |  |
| D3ZF13 | Ndufab1 | Acyl carrier protein | 2.76 | 0.01 |  |
| D3ZAF6 | Atp5j2 | ATP synthase subunit f, mitochondrial | 2.68 | 0.02 |  |
| Q6VPP3 | Clca4 | Chloride channel accessory 4 | 2.62 | 0.00 |  |
| Q5EGZ1 | Ace2 | Angiotensin-converting enzyme 2 | 2.52 | 0.00 |  |
| P06866 | Hp | Haptoglobin | 2.43 | 0.00 |  |
| P25235 | Rpn2 | Dolichyl-diphosphooligosaccharide--protein glycosyltransferase subunit 2 | 2.42 | 0.02 |  |
| P62138 | Ppp1ca | Serine/threonine-protein phosphatase PP1-alpha catalytic subunit | 2.42 | 0.02 |  |
| P62142 | Ppp1cb | Serine/threonine-protein phosphatase PP1-beta catalytic subunit | 2.42 | 0.02 |  |
| P63088 | Ppp1cc | Serine/threonine-protein phosphatase PP1-gamma catalytic subunit | 2.42 | 0.02 |  |
| O55159 | Epcam | Epithelial cell adhesion molecule | 2.40 | 0.02 |  |
| P11240 | Cox5a | Cytochrome c oxidase subunit 5A, mitochondrial | 2.38 | 0.00 |  |
| Q63584 | Tmed10 | Transmembrane emp24 domain-containing protein 10 | 2.35 | 0.02 |  |
| Q64319 | Slc3a1 | Neutral and basic amino acid transport protein rBAT | 2.23 | 0.01 |  |
| F1LPR6 | Ighm | Uncharacterized protein | 2.03 | 0.02 |  |
| P20788 | Uqcrfs1 | Cytochrome b-c1 complex subunit Rieske, mitochondrial | 2.01 | 0.00 |  |
| **Down-regulated** | |  |  |  |  |
| P18422 | Psma3 | Proteasome subunit alpha type-3 | 0.49 | 0.00 |  |
| G3V647 | Pdxk | Pyridoxal kinase | 0.46 | 0.02 |  |
| P36860 | Ralb | Ras-related protein Ral-B | 0.31 | 0.02 |  |
| Q6AYS3 | Ctsa | Carboxypeptidase | 0.25 | 0.02 |  |
| G3V7H6 | Arg2 | Arginase | 0.23 | 0.02 |  |
| Q8CHN5 | Npc2 | Epididymal secretory protein 1 | 0.19 | 0.01 |  |
| Q7TP52 | Cmbl | Carboxymethylenebutenolidase homolog | 0.18 | 0.01 |  |
| Q6PDW8 | Gpx1 | Glutathione peroxidase | 0.16 | 0.01 |  |
| P27615 | Scarb2 | Lysosome membrane protein 2 | 0.15 | 0.00 |  |
| Q6AXR4 | Hexb | Beta-hexosaminidase subunit beta | 0.13 | 0.01 |  |
| Q6MGA7 | RT1-DMb | Hla-dmb protein | 0.10 | 0.01 |  |
| P52759 | Hrsp12 | Ribonuclease UK114 | 0.10 | 0.00 |  |
| Q6P7S1 | Asah1 | Acid ceramidase | 0.08 | 0.00 |  |
| G3V844 | Amy2a3 | Alpha-amylase | 0.04 | 0.01 |  |

**Supplementary Methods**

**Antibiotics dose**

**Table S5** Conversion of animal doses to human equivalent doses based on the body surface area are as follow (http://www.lascn.com/teach/readteach.asp?id=68).

|  | Mouse  (20 g) | Rat  (200 g) | Cavy (400 g) | Rabbit (1.5 kg) | Cat  (2 kg) | Monkey (4 kg) | Dog  (12 kg) | Human (70 kg) |
| --- | --- | --- | --- | --- | --- | --- | --- | --- |
| Mouse (20 g) | 1.0 | 7.0 | 12.25 | 27.8 | 29.7 | 64.1 | 124.2 | 378.9 |
| Rat (200 g) | 0.14 | 1.0 | 1.74 | 3.9 | 4.2 | 9.2 | 17.8 | 56.0 |
| Cavy (400 g) | 0.08 | 0.57 | 1.0 | 2.25 | 2.4 | 5.2 | 4.2 | 31.5 |
| Rabbit (1.5 kg) | 0.04 | 0.25 | 0.44 | 1.0 | 1.08 | 2.4 | 4.5 | 14.2 |
| Cat (2 kg) | 0.03 | 0.23 | 0.41 | 0.92 | 1.0 | 2.2 | 4.1 | 13.0 |
| Monkey (4 kg) | 0.016 | 0.11 | 0.19 | 0.42 | 0.45 | 1.0 | 1.9 | 6.1 |
| Dog (12 kg) | 0.008 | 0.06 | 0.1 | 0.22 | 0.23 | 0.52 | 1.0 | 8.1 |
| Human (70 kg) | 0.0026 | 0.018 | 0.031 | 0.07 | 0.078 | 0.16 | 0.82 | 1.0 |

**Analysis of triazine herbicides in blood**

Triazine herbicides were quantified by HPLC-MS/MS with a C_18_ column (150 × 2.1 mm, 3 μm, Waters, Shanghai, China). Seventy percent acetonitrile and thirty percent water (containing 0.1% formic acid) were used as the the mobile phase and the flow rate was 0.3 mL/min. The injection volume was 10 μL. Setup for mass spectrometer were as follows: spray voltage of positive ionization mode 3000 V; vaporizer temperature 200 °C; sheath gas pressure 40 Arb; axu gas pressure 10 Arb; ion sweep gas pressure 0 Arb; collision gas pressure 1.5 mTorr. The specific selective reaction monitoring (SRM) was used and more instrument setup was presented in Table S6.

**Table S6** Parameters of moleculars fragmentation.

| Compounds | Parent Mass (m/z) | Product Mass (m/z) | Collision Energy (eV) |
| --- | --- | --- | --- |
| Simazine | 202.00 | 132.100 | 18 |
|  |  | 124.170 | 17 |
| Metribuzin | 215.054 | 187.06 | 17 |
|  |  | 130.98 | 22 |
| Ametryn | 228.06 | 186.048 | 17 |
|  |  | 96.124 | 25 |
| Atrazine | 216.027 | 174.015 | 17 |
|  |  | 104.108 | 31 |
| Terbuthylazine | 230.190 | 174.05 | 15 |
|  |  | 104.11 | 32 |
| Metamitron | 203.1 | 174.4 | 15 |
|  |  | 77.2 | 35 |

Method validation procedures included studies of linearity, recoveries, limit of detection (LOD), limit of quantification (LOQ), accuracy and precision. Recovery study was conducted by fortifying triazine herbicides standard into each blank blood sample at three levels. Three replicates were carried out at each spiking level to determine the average recovery and relative standard deviation (RSD). The linearity was investigated by calculating the correlation coefficients (r^2^) of analytical curve diluted at five different concentration levels. LOD and LOQ was respectively calculated at e signal-to-noise (S/N) of 3 and 10 via determining the minimum concentration of each compound of linear rang. As showed in Table, the average recoveries of the analytes were in the range of 87.63–106.4% and RSD values ranged from 1.77% to 24.15%.

**Table S7** Summary of average recovery, LOD and LOQ of triazine herbicides in blood (n=3).

| Compound | Recovery ± RSD (%, n=3) | | | LOD  μg/kg | LOQ  μg/kg |
| --- | --- | --- | --- | --- | --- |
|  | 10 μg/kg | 100 μg/kg | 500 μg/kg |  |  |
| Simazine | 95.22±2.73 | 97.86±12.41 | 93.27±13.98 | 0.03 | 0.1 |
| Ametryn | 91.74±1.77 | 98.47±13.19 | 95.26±13.11 | 0.01 | 0.04 |
| Metribuzin | 106.40±23.85 | 97.55±10.96 | 103.43±11.22 | 0.1 | 0.3 |
| Atrazine | 92.75±2.96 | 98.20±10.13 | 95.06±24.15 | 0.03 | 0.1 |
| Terbuthylazine | 87.63±4.69 | 94.73±11.91 | 94.72±23.20 | 0.03 | 0.1 |

**Table S8** The linear range and correlation coefficient (r^2^) in blood five levels (n=3).

| Herbicide | Linear range (μg/kg) | Linear equation | Correlation coefficients r^2^ |
| --- | --- | --- | --- |
| Simazine | 0.1-400 | y = 0.0299x + 0.0525 | 0.9998 |
| Ametryn | 0.04-10 | y = 0.2342x + 0.0124 | 0.9919 |
| Metribuzin | 0.8-1600 | y = 0.0195x + 0.5455 | 0.9955 |
| Atrazine | 0.4-400 | y = 0.1029x - 0.0869 | 0.9997 |
| Terbuthylazine | 0.1-100 | y = 0.1378x + 0.0352 | 0.9998 |

**In vitro microbial metabolism and microsomal metabolism of triazine herbicides**

**Analysis of triazine herbicides in microsomes**

An aliquot of 0.5 mL of microsomes solution and 1 mL of acetonitrile was vortexed for 3 min with 0.2 g of NaCl, and then centrifuged at 4000 *g* for 5 min. The supernatant was dried at 35 °C under a nitrogen atmosphere and adjusted to a final volume with 0.5 mL of acetonitrile containing 50 μg/L of metamitron (internal standard) to obtain a solution for HPLC-MS/MS (setup was the same with the herbicides analysis in blood).

**Analysis of triazine herbicides in microbial culture medium**

An aliquot of 1 mL of microbial culture medium and 2 mL of acetonitrile was vortexed for 3 min with 0.2 g of NaCl, and then centrifuged at 4000 *g* for 5 min. The supernatant was dried at 35 °C under a nitrogen atmosphere and adjusted to a final volume with 1 mL of acetonitrile for HPLC-MS/MS (setup was the same with the herbicides analysis in blood).

**Hepatic metabolic enzyme mRNA expression**

**Table S9** Real Time Quantitative PCR primer pairs for hepatic metabolic enzymes (in the 5’ - 3’ direction, β-actin was internal control).

| Gene | Forward | Reverse |
| --- | --- | --- |
| CYP1A2 | GTCACCTCAGGGAATGCTGTG | GTTGACAATCTTCTCCTGAGG |
| CYP2C11 | GGAGGAACTGAGGAAGAGCA | AATGGAGCATATCACATTGCAG |
| CYP2D2 | GAAGGAGAGCTTTGGAGAGGA | AGAATTGGGATTGCGTTCAG |
| CYP2E1 | ATCGCCACCCTCCTCCTCGTAT | AGCACCTCCTTGACAGCCTTGT |
| CYP3A1 | GCCTTTTTTTGGCACTGTGCT | GCATTTGACCATCAAACAACCC |
| CYP3A2 | GCTTTCAGCTCTCACACTGGAAA | TCTATGGGTTCCAAGTCGGTAGA |
| CYP3A11 | ACAAACAAGCAGGGATGGAC | CCCATATCGGTAGAGGAGCA |
| GSTYc2 | AAGCTGAGCAGGGCTGATGT | ACAATGCCTGGGTCCATCTC |
| GSTM2 | CACGCCATTCGCCTGTTCCT | GGTGTCCATAGCCTGGTTCTCC |
| β-actin | ACTATCGGCAATGAGCGGTTCC | AGCACTGTGTTGGCATAGAGGT |

**Label-free proteomics of the small intestinal mucosa**

**Table S10** The elution gradient used in proteins separation.

| Time (min) | %A | %B |
| --- | --- | --- |
| 0 | 95 | 5 |
| 5 | 92 | 8 |
| 35 | 85 | 15 |
| 60 | 70 | 30 |
| 80 | 55 | 45 |
| 100 | 20 | 80 |
| 110 | 20 | 80 |
| 110.1 | 95 | 5 |
| 120 | 95 | 5 |
